# Dual Delayed Feedback Provides Sensitivity and Robustness to the NF-*κ*B Signaling Module

**DOI:** 10.1371/journal.pcbi.1003112

**Published:** 2013-06-27

**Authors:** Diane M. Longo, Jangir Selimkhanov, Jeffrey D. Kearns, Jeff Hasty, Alexander Hoffmann, Lev S. Tsimring

**Affiliations:** 1 Department of Bioengineering, University of California San Diego, La Jolla, California, United States of America; 2 San Diego Center for Systems Biology, La Jolla, California, United States of America; 3 Department of Chemistry and Biochemistry, University of California San Diego, La Jolla, California, United States of America; 4 Molecular Biology Section, Division of Biological Sciences, University of California San Diego, La Jolla, California, United States of America; 5 BioCircuits Institute, University of California San Diego, La Jolla, California, United States of America; Princeton University, United States of America

## Abstract

Many cellular stress-responsive signaling systems exhibit highly dynamic behavior with oscillatory features mediated by delayed negative feedback loops. What remains unclear is whether oscillatory behavior is the basis for a signaling code based on frequency modulation (FM) or whether the negative feedback control modules have evolved to fulfill other functional requirements. Here, we use experimentally calibrated computational models to interrogate the negative feedback loops that regulate the dynamic activity of the transcription factor NF-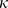
B. Linear stability analysis of the model shows that oscillatory frequency is a hard-wired feature of the primary negative feedback loop and not a function of the stimulus, thus arguing against an FM signaling code. Instead, our modeling studies suggest that the two feedback loops may be tuned to provide for rapid activation and inactivation capabilities for transient input signals of a wide range of durations; by minimizing late phase oscillations response durations may be fine-tuned in a graded rather than quantized manner. Further, in the presence of molecular noise the dual delayed negative feedback system minimizes stochastic excursions of the output to produce a robust NF-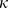
B response.

## Author Summary

Many signaling events are controlled by negative feedback circuits: as a result they are highly dynamic and in some cases show oscillations The presence of oscillations has led to the hypothesis that signaling pathways convey information about the stimulus via the frequency of oscillations and spikes of activity, analogous to frequency modulated (FM) radio signals. One such signaling protein is NF-kB which controls the inflammatory and immune response to cytokines and pathogens. We show here that the topology of the negative circuit does not allow for frequency modulation by the signaling input. Instead, we show that a second negative feedback circuit may be tuned to dampen the oscillations. In fact, the resulting dual negative feedback motif allows for better tracking of the duration of the incoming signal than the single negative feedback circuit, as well as better buffering of noise present in the incoming signal. Thus we propose that the negative feedback topology has evolved to provide complex dynamics of NF-kB in vertebrate animals and not for the purposes of oscillations.

## Introduction

Many important signal transduction pathways contain a negative feedback motif consisting of an activator that activates its own repressor. Activated repression is capable of generating oscillatory behavior [1] and has been observed to do so in biological systems such as the Hes1 regulatory protein which controls neuronal differentiation [2], the p53-Mdm2 system that mediates the DNA damage response [3], and the NF-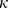
B (Q04207) signaling network that governs the immune response and inflammation [4], [5].

The role of activated repression is well understood in the context of transient signaling as functioning to limit the duration of the induced activity. Indeed, misregulation of the negative feedback mechanisms that control NF-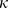
B and p53 has been shown to generate prolonged inflammatory or genotoxic stress responses, respectively, that lead to cell death or chronic disease [6], [7]. Further, negative feedback can sensitize and speed-up responses to weak or transient input signals [8] when compared to constitutive attenuation mechanisms.

In contrast, the physiological role of oscillatory signaling behavior remains poorly understood. Recent work has shown that, in the calcium stress pathway in yeast, the frequency of nuclear localization of a stress-response transcription factor can be modulated by the magnitude of the extracellular calcium concentration, and this frequency modulation results in a coordinated expression of target genes [9]. In the NF-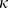
B and p53 signaling systems, the function of oscillations is still unknown. Oscillations in p53 activity were proposed to represent a counting mechanism that quantizes the response, ensuring a robust but appropriate amount of activity for a specific degree of DNA damage [10]. An alternate view was proposed in which oscillations of the p53-controlling ATM kinase activity allow for periodic sampling of the damaged DNA to track its repair and, if necessary, drive further p53 signaling to sustain the repair programs [11]. Oscillations in NF-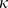
B activity were proposed to determine which genes would be transcriptionally induced, thereby representing a temporal code that conveys information about the stimulus to gene promoters [5]. However, it is not clear whether or not the frequency encodes information in this systems as no differences in NF-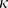
B target gene expression were observed between oscillating and non-oscillating genetic variants [12].

Recent work has demonstrated that oscillations in NF-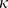
B activity can be generated by pulsatile stimulation with TNF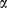 (P06804) [13]. However, an analysis of the repeated activation of NF-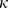
B that is driven by an oscillating signal provides little information about the role of oscillations that naturally arise with persistent stimulation. Thus, the role(s) of oscillations in NF-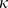
B activity remains unclear and several questions are still unanswered: Do these oscillations convey information encoded in the frequency to downstream processes? Do they function to generate a periodically recurring phase of sensitivity to stimuli or regulatory crosstalk representing a potential “counting” mechanism? Do they “quantize” the output signal, thus specifying robust units of activity? Or, are the oscillations caused by persistent signaling simply a non-functional by-product of the requirement for the negative feedback architecture to enable sensitive, fast responses to transient stimuli?

Mathematical models comprised of a small number of equations have led to a greater understanding of biological processes in terms of molecular interactions, diffusion, dose responses, gradient sensing, the role stochasticity in gene expression and in fate decisions [14]–[17]. Although several models of networks with autoregulation have been developed [18]–[20], most of these networks do not incorporate delays. In signaling, however, such elegant models often do not faithfully reproduce the dynamic behavior of the signaling system because actual biological networks involve many molecular interactions that tend to slow overall signal processing. Larger models comprised of many molecular species and parameters have proven useful in exploring dynamic signaling behavior via computational simulations in conjunction with experimental studies, but they are analytically intractable and therefore do not provide the degree of conceptual insights that small models do.

Here we pursue an alternative approach to modeling NF-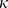
B signaling. We construct a new model that replaces cascading reactions with a single but delayed compound reaction that enables both recapitulation of experimentally observed dynamics and the use of powerful analytical tools. With these tools, we explore the physiological function of the dynamic behavior of NF-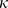
B produced by the activated repression mechanism mediated by its inducible inhibitors, I
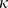
B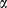 (Q9Z1E3) and I
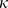
B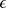 (O54910). The mathematical analysis results in predictions that are addressed experimentally and thus lead to fundamental insights about the function and origins of this signaling system.

## Results

### NF-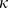
B model formulation

**Figure 1:**
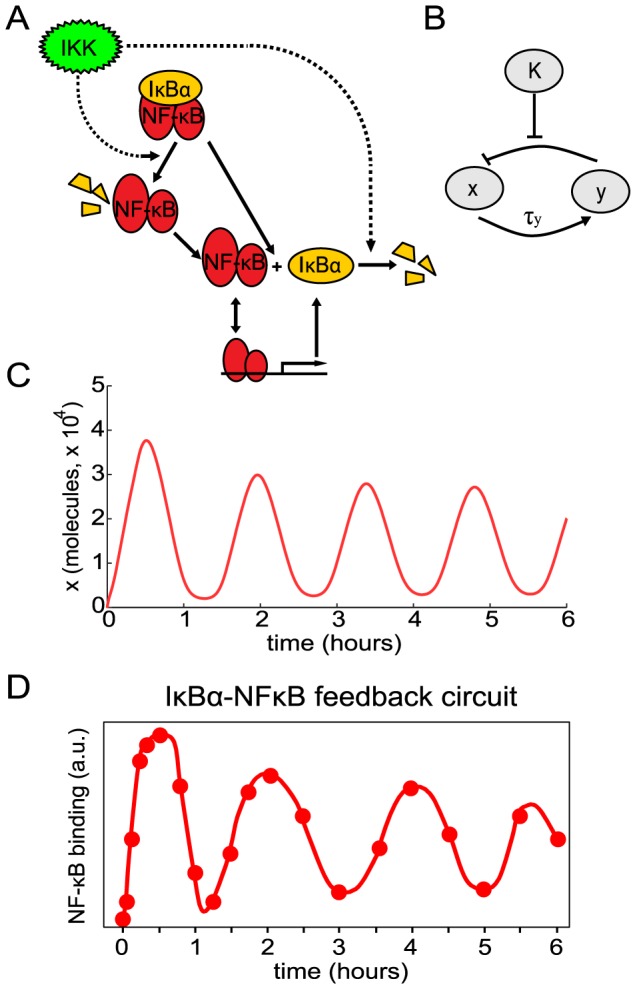
Oscillatory behavior from a system with a single negative feedback loop. (A) Diagram of the I
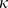
B
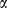
- NF-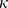
B signaling module. (B) Diagram of a system with a single delayed negative feedback loop. (C) Nuclear NF-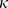
B levels (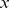) in response to persistent stimulation as a function of time produced using our delayed feedback model. (D) Experimental levels of nuclear NF-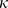
B (determined by EMSAs) in cells with only the I
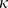
B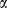-mediated negative feedback loop intact (data from [4]).

The basic structure of the NF-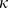
B signaling module is shown in Figure 1 *A* [4]. In resting cells, NF-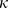
B is sequestered in the cytoplasm by I
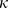
B proteins. Cellular stimulation leads to activation of the I
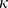
B kinase (IKK) which phosphorylates I
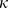
B proteins thus targeting them for degradation. Upon degradation of I
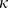
B proteins, NF-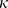
B moves into the nucleus and activates hundreds of target genes including the predominant I
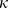
B isoform, I
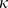
B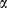. Synthesized I
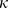
B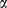 enters the nucleus, binds to NF-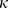
B, and the I
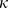
B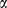-NF-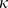
B complex is exported back to the cytoplasm. Thus, the core feature of the NF-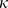
B signaling module is a negative feedback loop mediated by I
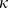
B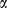. This can be abstracted to a simple motif in which 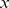 (NF-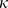
B) activates 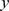 (I
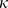
B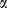), 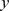 represses 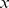, and repression of 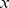 by 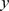 is relieved by 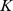 (active IKK) (Figure 1*B*).

Using this motif as a guide, we formulated our model of the I
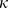
B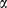-mediated NF-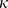
B response as a set of 9 reactions and 6 variables (Tables 1, 2). Specifically, the model assumes that the total number of the NF-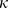
B molecules (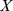) is conserved, however they can exist either in free/nuclear form (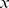) or sequestered outside of nucleus within the I
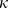
B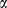-NF-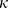
B complex (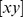). The model contains non-delayed reactions for the binding of free NF-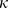
B to the unbound I
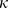
B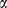 promoter (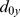) to form the bound I
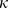
B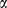 promoter (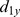), binding of I
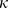
B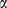 protein (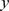) to free NF-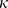
B to form the I
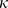
B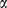-NF-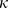
B complex, constitutive degradation of I
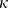
B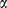, and induced degradation of free and bound I
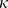
B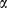 proteins by the active I
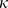
B kinase IKK (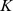) producing free NF-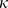
B. In contrast, a compound delayed reaction describes the synthesis of I
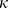
B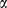 protein. This reaction involves a time delay 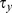, which represents the time needed for transcription, translation, nuclear import and export, and protein-protein interactions.

**Table 1:** Single feedback model variables.

Variable	Description
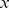	nuclear NF- 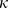 B (nM)
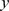	free I 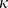 B 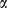 (nM)
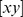	NF- 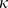 B -bound I 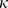 B 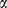 (nM)
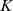	active I 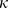 B kinase (IKK)
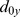	unbound I 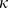 B 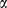 promoter*
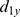	NF- 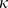 B -bound I 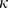 B 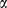 promoter*

* average numbers of corresponding promoters.

**Table 2:** Single feedback model reactions.

Reaction	Rate	Description
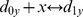	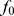 , 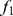	NF- 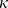 B binds (and unbinds) I 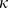 B 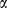 promoter
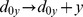	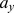	constitutive synthesis of I 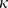 B 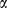 (delayed reaction)
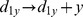	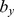	induced synthesis of I 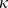 B 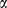 (delayed reaction)
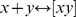	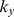 , 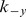	I 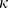 B 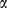 association (and dissociation) with NF- 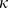 B
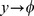	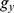	constitutive degradation of I 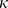 B 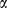
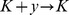	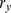	IKK-mediated degradation of I 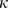 B 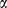
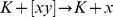	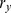	IKK-mediated degradation of NF- 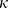 B-bound I 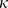 B 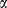

Using experimentally validated assumptions, we reduced the set of mass-action kinetics equations for the 9 reactions to a single delay-differential equation:
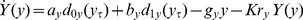
(1)where 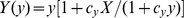 is the total I
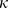
B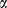 concentration (the sum of free I
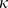
B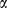 (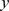) and I
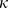
B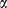 bound to NF-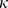
B), 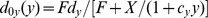, 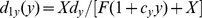, are the probabilities for the I
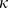
B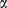 promoter to be free or bound to NF-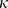
B, respectively, 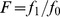, 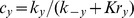, and the subscript 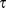 denotes the variable taken at time 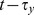 (see Methods for details of the derivation). The rates of individual reactions 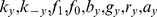 are defined in Table 2.

Mirroring the biological system, the non-dimensional time-dependent parameter 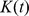, which characterizes the active IKK concentration, is used as the proxy input signal. The first term in the r.h.s. of Eq. 1 represents constitutive synthesis from the unbound I
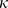
B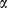 promoter, the second term represents induced synthesis from the NF-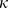
B-bound I
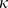
B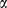 promoter, the third term represents constitutive degradation of I
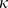
B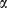 protein, and the fourth term represents IKK-induced degradation of I
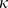
B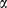. Values of 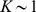 correspond to the rate of IKK-induced degradation of NF-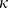
B-I
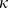
B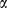 complex which is of the same magnitude as unbound I
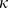
B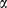. Nuclear NF-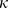
B level 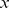 at any time can be determined directly from I
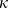
B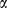 levels via 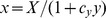. The time delay 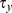 is incorporated in the synthesis terms: we assume that the rate of production of new proteins at time 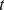 depends on the state of the system at time 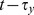. Incorporating this time delay allows us to explore the behavior of the negative feedback loop without simulating the full set of reactions associated with it. We obtained values for the time delay and for the other model parameters by calibrating the behavior of the model with experimental results (Table S1). As a starting point, we used parameter values from biochemical measurements [21]. However, some modifications were necessary because these values represent the rates of single reaction steps and the model contains compound reactions.

To validate the model, we compared it to experiments. In response to a persistent input signal (starting at time 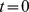), our simulations of the I
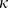
B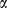-mediated negative feedback system show pronounced oscillations in nuclear NF-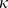
B levels with an oscillation period of about 90 minutes (Figure 1 *C*). Oscillations with a similar period were observed experimentally when mutant cells containing only the I
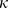
B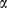 feedback loop were persistently stimulated with the inflammatory cytokine TNF (Figure 1*D*).

To address the dynamics of the wild-type NF-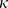
B system that feature both I
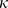
B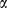 and I
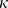
B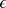 feedback loops, we expanded the model to include an additional 9 reactions and 4 variables involving I
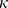
B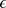 (Tables 3, 4). Following the same reduction procedure (see Methods for derivation), we derived a deterministic model consisting of two coupled delay-differential equations for the concentrations of the two I
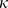
B isoforms, I
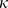
B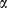 (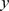) and I
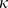
B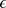 (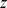),
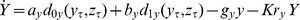
(2)

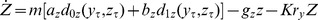
(3)where 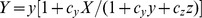, 
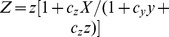
, 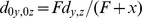, 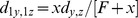, 
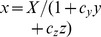
, 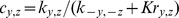, and 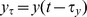, 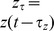. Parameter 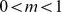 here is the scaling factor which characterizes the relative strength of the secondary feedback loop.

**Table 3:** I
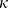
B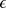 feedback model variables.

Variable	Description
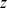	free I 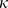 B 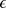 (nM)
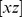	NF- 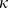 B -bound I 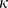 B 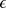 (nM)
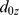	unbound I 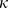 B 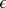 promoter*
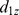	NF- 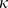 B -bound I 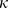 B 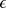 promoter*

* average numbers of corresponding promoters.

**Table 4:** I
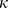
B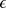 feedback model reactions.

Reaction	Rate	Description
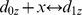	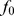 , 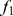	NF- 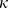 B binds (and unbinds) I 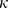 B 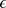 promoter
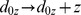	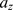	constitutive synthesis of I 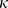 B 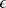 (delayed reaction)
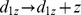	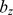	induced synthesis of I 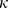 B 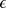 (delayed reaction)
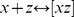	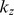 , 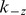	I 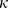 B 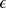 association (and dissociation) with NF- 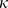 B
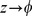	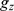	constitutive degradation of I 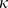 B 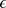
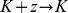	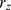	IKK-mediated degradation of I 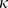 B 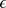
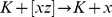	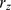	IKK-mediated degradation of NF- 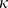 B-bound I 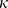 B 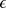

In Eqs. 2 and 3, 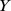 represents total I
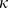
B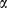 (the sum of free I
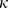
B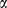 (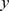) and I
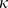
B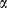 bound to NF-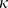
B (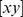), and 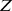 represents total I
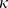
B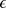 (the sum of free I
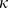
B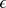 (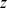) and I
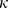
B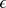 bound to NF-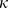
B (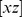)). The terms in the r.h.s. of Eqs. 2 and 3 again represent constitutive synthesis from the identical unbound I
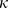
B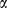 and I
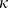
B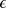 promoters, induced synthesis from the NF-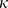
B-bound promoters, constitutive degradation of I
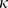
B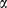 and I
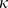
B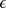 proteins, and IKK-induced degradation of I
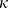
B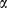 and I
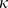
B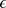. Nuclear NF-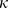
B levels are determined directly by I
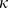
B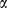 and I
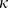
B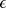 levels. Parameter values for the I
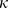
B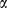-mediated reactions were determined in the previous section. For the I
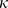
B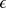 feedback reactions, we use the same parameter values except for the constitutive synthesis and the constitutive degradation rates, which were chosen based on experimental measurements [21] (Table S1).

### Is the oscillation period a function of the stimulus?

The advantage of our modeling approach is that it allows for analytical studies of the network dynamics. Here, we perform a linear stability analysis of the delay-differential equation (1) to identify the characteristic period and decay rate of NF-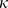
B oscillations produced when input signal is present (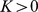). For sufficiently large 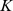, induced synthesis and degradation are much stronger than basal ones, so the latter can be neglected (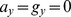).

**Figure 2:**
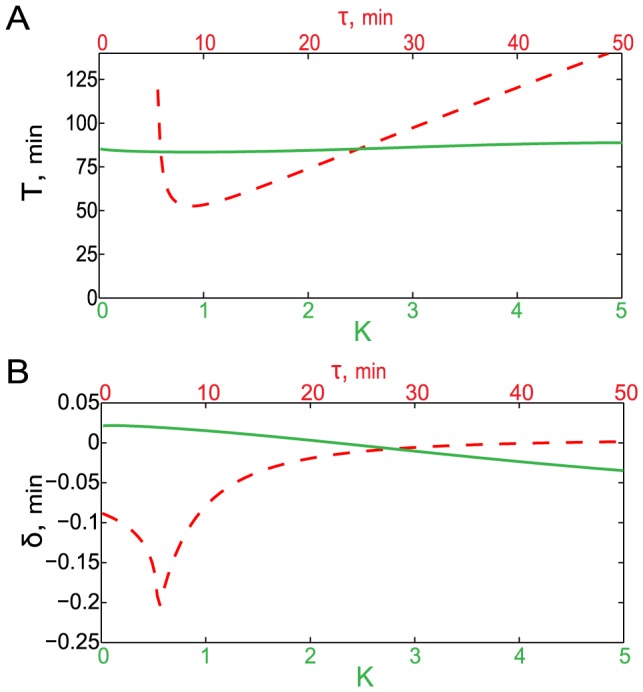
Period and decay rate of oscillations produced by the I
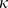
B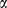-mediated negative feedback system. (A) The oscillation period 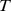 as a function of 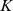 with 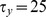 min (green line) and as a function of 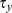 with 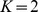 (red dashed line). (B) The oscillation decay rate 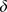 as a function of 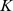 with 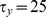 min (green line) and as a function of 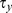 with 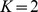 (red dashed line).

Expressing 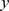 via 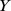 and substituting it into 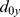, 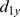 yields a closed equation for 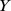 in the form
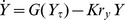
(4)where 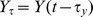 and the function 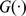 has the form
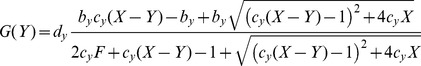
(5)The fixed point 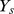 (stationary solution) of this equation is given by the algebraic equation
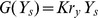
(6)The stability of this solution is determined by the eigenvalue of the linearized equation (4) linearized near the fixed point 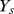 (see Methods for details). The corresponding eigenvalue can be found in terms of the Lambert function 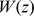 defined via 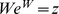,
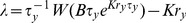
(7)The imaginary part of 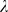 gives the oscillation frequency 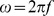, and the (negative) real part of 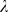 gives the decay rate 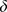 of oscillations. Plotting the period (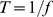) (Figure 2*A*) and decay (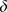) (Figure 2*B*) of the oscillations as a function of the delay reveals a strong dependence. In contrast, the signaling perturbation 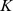 (the active IKK kinase) that acts as the input for the model determines the amplitude of the response but only negligibly affects the period or the oscillation decay (Figure 2*B*). The mathematical reason for this asymmetry is that the imaginary part of the Lambert function 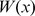 for negative values of its argument changes very weakly for arguments below 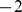 (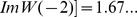, 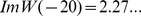) and asymptotically approaches 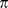 for very large negative values of the argument. This is why the period of dampened oscillations (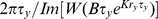) depends strongly on delay 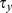 and only very weakly on 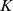. Meanwhile, the real part of the eigenvalue 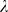 (the decay rate) is linearly proportional to 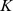 because of the second term in Eq.(7) and also strongly depends on 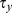 because of the first term. Thus, we find that the period is highly dependent on the delay but is rather insensitive to changes in the input level. This is confirmed by direct simulations of the full nonlinear equation (1), where time series of 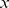 are plotted for several different values of 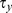 and 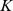 (Figure S1). Since variations of stimulus do not lead to significant frequency modulation of NF-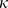
B activity, oscillations of NF-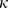
B are unlikely to encode information about the stimulus.

### Damping of oscillations in a dual delayed feedback loop system

**Figure 3:**
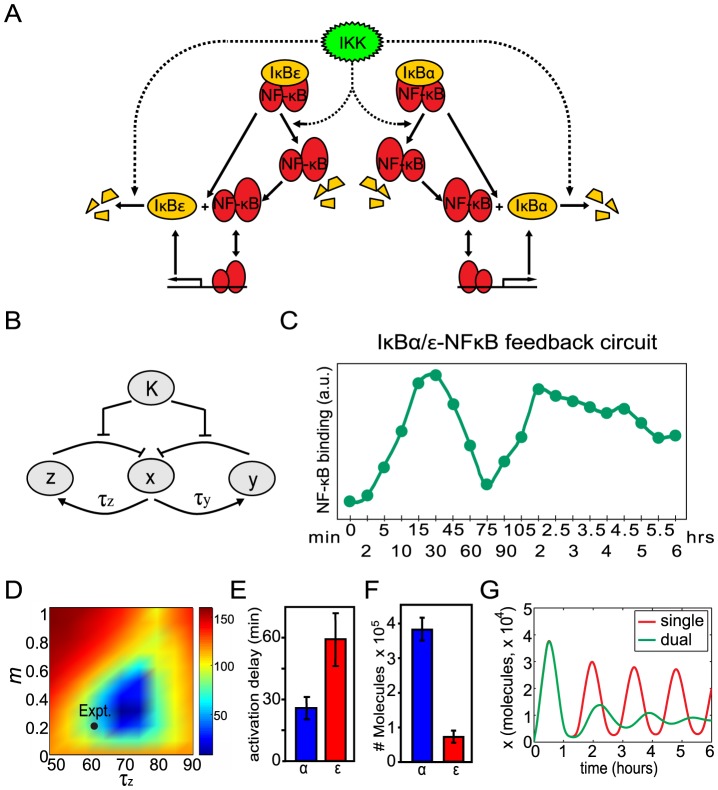
Damped oscillations with a dual negative feedback system. (A) Diagram of the dual feedback system with both I
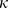
B
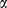
- and I
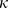
B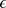-mediated negative feedback loops. (B) Diagram of a system with dual delayed negative feedback loops. (C) Experimental levels of nuclear NF-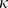
B (determined by EMSAs) in wild-type cells containing both I
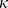
B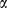-and I
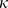
B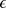-mediated negative feedback (data from [17]). (D) Optimization of the parameters of the second feedback loop 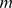 and 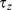 towards maximizing the oscillations damping. The optimization method minimizes peak-minus-trough differences six hours after the onset of stimulation, the global minimum occurs at 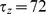 min, 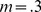. The black dot indicates the experimentally measured parameter values (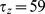 min, 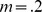). Note that 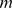 was not measured directly. The value of 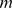 corresponding to the experimentally measured value of 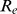 was determined with the model (Figure S3). (E) Experimental measurements of I
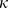
B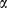 and I
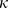
B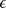 synthesis delays. (F) Experimental values for peak I
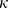
B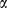 and I
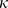
B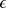 protein levels. (G) Simulated time course of nuclear NF-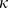
B levels (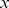) for the single feedback system and for the optimized dual feedback system in response to persistent stimulation with 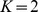.

The main qualitative difference between the one-loop system considered in the previous section, and the wild-type NF-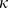
B module is the presence of another I
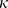
B isoform, I
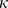
B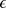, which also provides negative feedback regulation on NF-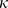
B activity (Figure 3*A, B*), however with slower kinetics [21]. Experimental and computational work has shown that I
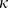
B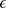-mediated feedback can cause damping of I
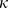
B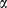 -mediated oscillations [21] and (Figure 3*C*). More recent computational work has predicted that I
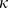
B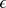-mediated feedback desynchronizes oscillations but does not dampen oscillations in single cells [13]. Thus, the mechanism by which I
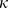
B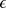-mediated feedback produces damped oscillations at the population level is not well established. Furthermore, it is unknown whether the damping function of the I
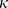
B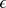-mediated feedback loop has evolved to achieve a specific regulatory function or may simply be a secondary consequence of another function. We hypothesize that the primary role of the second feedback loop is to mitigate oscillatory behavior produced by the first feedback loop.

To address our hypothesis that I
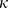
B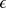-mediated feedback specifically evolved to dampen I
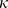
B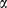-mediated oscillations, we performed a parameter optimization procedure on the wild-type model (Eqs. 2 and 3) to determine the I
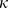
B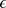 synthesis parameters that result in maximum damping. To characterize the degree of damping, we chose the maximum peak-trough difference after 6 hrs as a metric for the persistence of oscillations. According to the definition of this performance metric, “optimal damping” occurs when this metric is minimized. In our optimization procedure, we varied two important parameters, the time delay of the second feedback loop 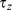 and the scaling factor 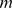 which simultaneously varies the rates of constitutive and induced synthesis of I
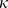
B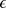. Choosing 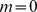 is equivalent to the complete removal of the I
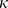
B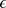-mediated negative feedback loop while 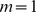 represents the case in which the inducible synthesis rates for I
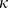
B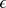 are the same as for I
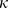
B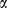. The two-dimensional optimization search is shown in a color map (Figure 3*D*) indicating that the performance metric is minimized at 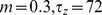. Time course simulations with the optimized parameter set show a high degree of damping (Figure 3*G*) similar to what is observed experimentally (Figure 3*C*).

To determine whether these optimized parameter values correspond to observations, we measured relevant parameter values experimentally. The synthesis delays for I
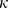
B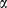 and I
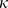
B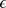 were determined by measuring I
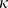
B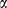 and I
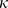
B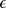 mRNA levels in a time course of TNF-treated murine embryonic fibroblasts (MEFs) in multiple independent experiments (Figure 3*E*, S2 *A,B*). The measured delay for I
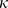
B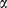 was 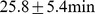, and 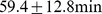 for I
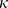
B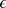, which agrees well with the model prediction for optimal damping.

Since it is difficult to measure the promoter strength experimentally, we employed an implicit way of comparing experiment with the model. To relate the parameter value 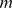 to experimental measurements, we set 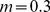 in the model and calculated the ratio of peak values for I
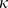
B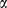 and I
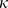
B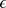 proteins 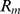, which we found to be equal 3.9. Then we measured the ratios of basal (unstimulated) to peak protein levels for I
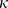
B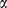 and I
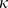
B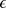 in experiment via quantitative Western blots of whole cell lysates generated during a TNF time course. These were compared to recombinant protein standards to derive absolute molecule number per cell. Peak I
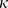
B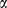 protein levels were measured to be 379,800 molecules per cell, and I
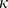
B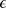 71,300 molecules per cell, with both values being subject to an estimated 25% error (Figure 3*F*, S2 *C,D*). These protein levels correspond to the experimental peak values ratio 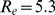 which is close to the model prediction 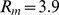.

### Duration encoding in a dual delayed negative feedback loop system

**Figure 4:**
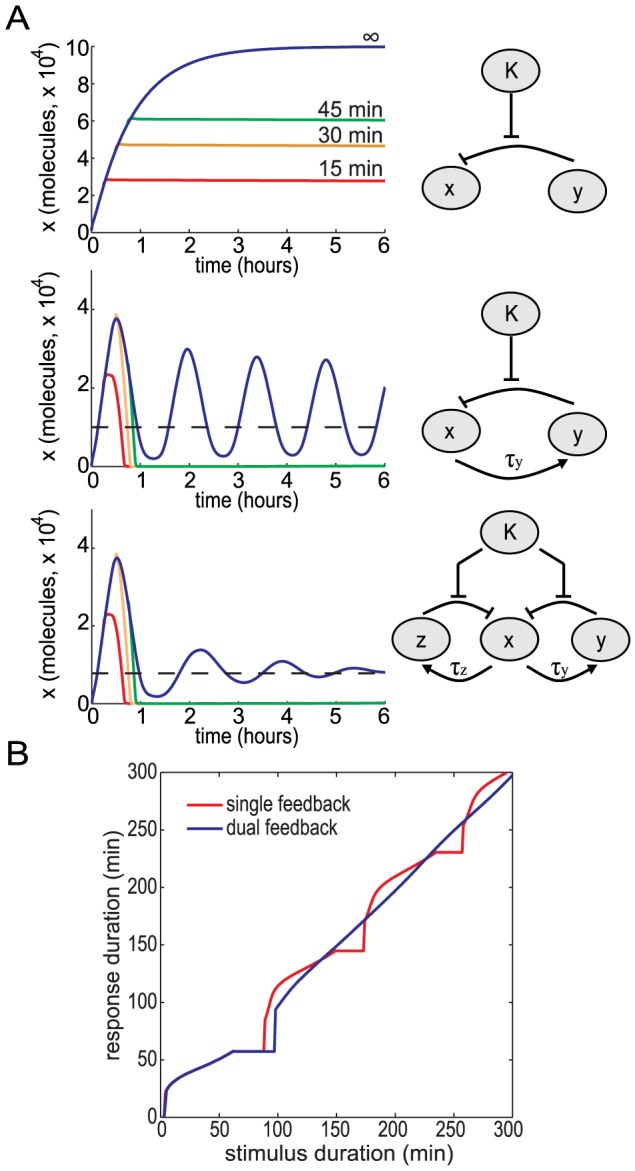
Response of the NF-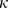
B signaling module to transient inputs with magnitude 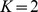, (A) Time series of 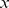 for a system with all feedback removed (top), a system with I
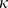
B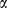-mediated negative feedback (middle), and a system with both I
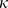
B
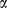
- and I
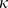
B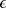-mediated negative feedback (bottom) in response to 15 min (red), 30 min (orange), 45 min (green), and persistent (blue) stimulation. (B) The response duration as a function of the stimulus duration for the single feedback and dual feedback systems. The response duration is the amount of time 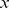 exceeds a threshold level of 50 (as indicated by the dashed black lines in the graphs shown in (A).

We next addressed why the NF-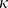
B signaling module may have evolved to produce oscillatory behavior if the oscillation frequency is not a function of the stimulus and does not constitute a signaling code. We first simulated persistent stimulation of a variant NF-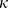
B system without feedback (we assume that I
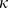
B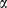 is constitutively produced, so 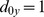, 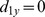 in Eq. 1) and found that this system produces long term, non-oscillatory NF-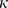
B activity (Figure 4*A* *Top, blue line*). As TNF is secreted in bursts and therefore perceived by surrounding cells as transient or pulse stimulation, we then performed stimulations of pulses 15, 30, and 45 min in duration. In the negative feedback-deficient NF-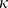
B system, the pulses resulted in transient responses that were attenuated very slowly. Faster attenuation can be achieved by increasing the constitutive synthesis rate, 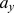. Increasing 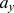 by two orders of magnitude results in pulse NF-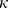
B responses to transient stimuli, but the responsiveness (in amplitude) is much reduced (Figure S4).

We then performed similar simulations in a single negative feedback loop NF-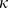
B system and found that this network topology allows for a rapid shutdown of NF-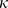
B activity for transient inputs (Figure 4*A* *Middle*). This suggests that the NF-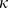
B network may have evolved from a pathway without feedback to a pathway with a single negative feedback loop to allow for a more sensitive transient response. Although the negative feedback indeed allows for greater sensitivity, a secondary consequence is that pronounced oscillations arise when the input signal persists for a long time period (Figure 4*A* *Middle, blue line*). The addition of a second negative feedback loop with a different time delay can help to dampen these oscillations, while preserving the responsiveness of the signaling module to transient stimuli (Figure 4*A* *Bottom*).

By plotting the duration of the response (above a given threshold) we investigated what may be called “temporal dose response curves” of the single and dual feedback systems (Figure 4*B*). The dual feedback system has a response duration close to 60 min for short pulses (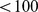 min), and a duration proportional to the input duration for longer pulses. The single feedback system has the same behavior as the dual feedback system for short inputs, but for longer inputs the single feedback system produces a quantized response with the same output duration for several different input durations. Our analysis indicates that a dual feedback system is able to produce temporally graded responses, whereas a single feedback system that oscillates does not. Given that the duration of the second phase of the NF-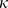
B response to TNF is a critical determinant of gene expression programs [4], we suggest that the NF-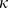
B system has evolved a dual feedback system that allows for NF-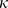
B activity whose duration is more closely related to the duration of the cytokine stimulus.

**Figure 5:**
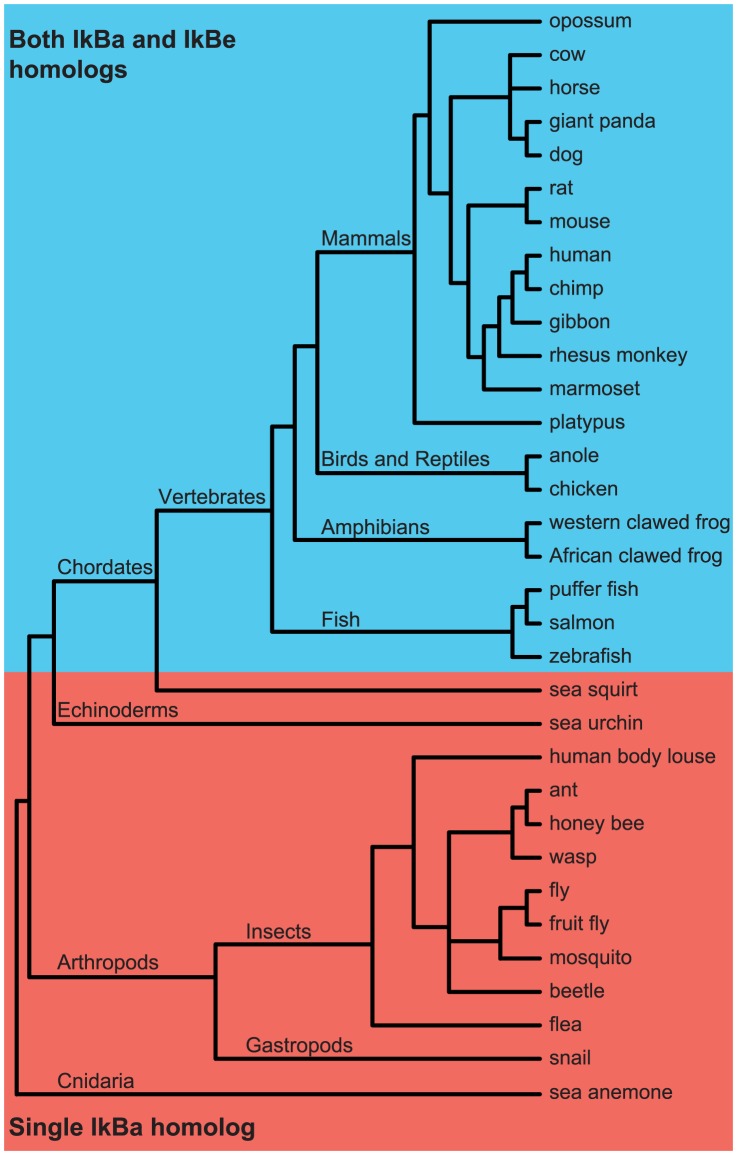
Schematic of a phylogenetic tree showing organisms in which I
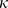
B homologs were found using BLASTP. Organisms with homologs for both I
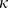
B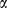 and I
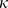
B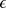 are in blue shaded region and organisms with a single homolog are in red shaded region. The branches in the schematic phylogenetic tree are not drawn to scale. (For simplicity, not all organisms with single or dual homologs are shown here. A complete list is provided in Table S6).

This fine temporal control, achieved via dual negative feedback, may be critical for complex cytokine-mediated cell-to-cell interactions involved in the adaptive immune response present in vertebrates, but may not be necessary for innate patogen-induced immune responses. We hypothesized that, on an evolutionary timescale, the appearance of dual negative feedback loops that regulate NF-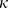
B activity may coincide with the transition from an innate to an adaptive immune system. To address this hypothesis, we used BLASTP with an E-value cutoff of 1e-25 to search for homologs of the mouse I
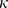
B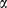 and I
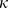
B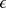 protein sequences in other organisms (see Methods). We found homologs for both I
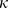
B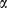 and I
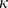
B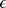, not only in other mammals (such as chimp, dog, platypus), but also in other vertebrate classes including fish, amphibians, and birds (Figure 5). Thus, dual negative feedback regulation of NF-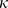
B activity appears to be present in all organisms with adaptive immunity. In contrast, we did not find any invertebrate organisms with homologs for both I
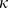
B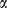 and I
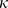
B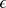 (Figure 5). Therefore, invertebrates, which lack adaptive immunity, also appear to lack the potential for dual negative feedback regulation of NF-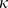
B mediated by I
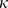
B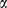 and I
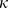
B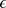 suggesting that the temporal control achieved with this regulatory architecture is not necessary for innate immune responses.

### Robustness to fluctuations in a dual delayed negative feedback loop system

Thus far, we have examined the response of the network to transient stimulation in the absence of fluctuations. However, it is well known that noise in gene expression can cause significant variability in cellular responses [18], [22]–[26]. Sometimes this variability can be beneficial [27], but in most cases, noise has a detrimental effect on the robustness of cellular functions. Mechanisms have presumably evolved to mitigate the unwanted effects of noise, especially in signaling pathways. In this section we examine the variability in the response of the NF-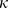
B module that arises due to intrinsic and extrinsic noise, and we demonstrate that the dual-feedback loop architecture allows for a more robust response than the single feedback loop system. Further, we investigate how the relative contribution of intrinsic and extrinsic fluctuations depends on the size of the system.

The concentration of signaling molecules such as NF-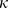
B can vary significantly between cells [28]. This variability in protein levels represents a source of extrinsic noise. We examined the variability in the response of the network to fluctuations in the total level of NF-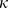
B and fluctuations in the IKK input level by simulating the network behavior with total NF-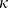
B levels and active IKK levels distributed within a certain rage around their nominal values. The coefficient of variation (CV) in peak nuclear NF-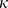
B levels and the CV in late-phase nuclear NF-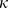
B levels is defined as 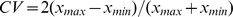 where 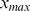 (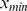) are the maximum (minimum) values of NF-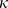
B at the peak or during the late phase. NF-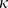
B late-phase response is defined as the nuclear NF-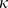
B level following the trough after the first peak response. In Text S1 we compare the extrinsic CV in the peak and the late phase for various values of IKK and NF-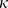
B (see Figure S5).

Intrinsic noise arises from the stochastic nature of biochemical processes such as transcription and translation [24]. To examine the response of the NF-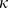
B signaling module in the presence of intrinsic genetic noise, we used the Gillespie algorithm [29] modified according to [30] to perform stochastic simulations of both regular and delayed biochemical reactions included in our delayed feedback model. These latter reactions are initiated at times dictated by their respective rates, but the numbers of molecules are only updated after the time delay since the reaction initiation.

**Figure 6:**
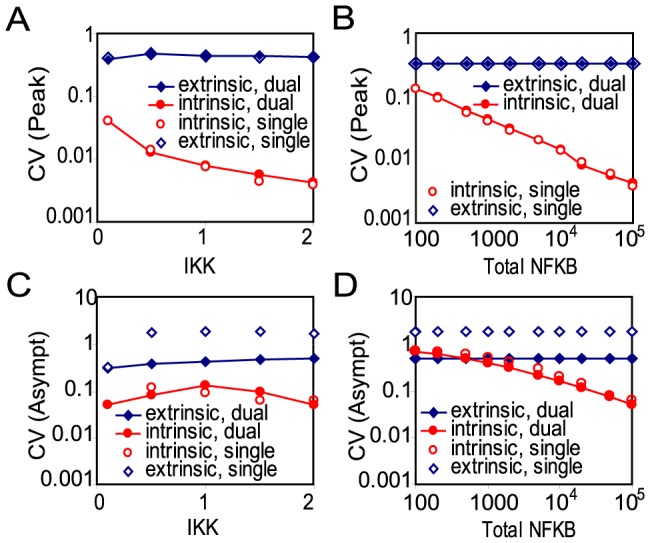
The coefficient of variation (CV) in nuclear NF-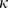
B levels due to extrinsic and intrinsic fluctuations. The CV was calculated for peak (A,B) and late-phase (C,D) nuclear NF-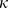
B levels for both single and dual feedback systems. The CV due to intrinsic fluctuations was determined from at least 50 runs of the stochastic simulations at each value of IKK (A,C) and total NF-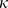
B (B,D). The CV due to extrinsic fluctuations in total NF-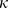
B and IKK levels was determined by varying the total NF-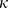
B level by 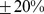 for each value of IKK (A,C) and by varying IKK by 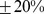 for value of total NF-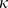
B (B,D).

We ran stochastic simulations of both a single and dual feedback system and estimated the ensemble average 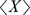 of the number of NF-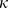
B molecules 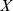 and the magnitude of fluctuations as characterized by the standard deviation 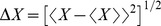 and the coefficient of variation 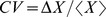. To determine how the variability in the response varies with the magnitude of the input and the size of the system, we determined the CV in peak nuclear NF-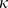
B levels and the CV in late-phase nuclear NF-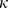
B levels for several values of IKK (Figure 6*A,C*) and for systems with up to 100,000 NF-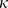
B molecules (Figure 6*B,D*). In Figure 6, we also plot CV values for extrinsic variations (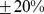) in total NF-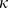
B at several values of IKK (Figure 6*A,C*) and CV values for extrinsic variations in IKK (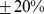) for several different system sizes (Figure 6*B,D*). We find that, even with this relatively low level (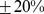) of extrinsic variability in IKK and NF-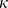
B protein levels [28], variability in the response of the network is dominated by extrinsic noise for large systems (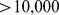 NF-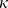
B molecules).

**Figure 7:**
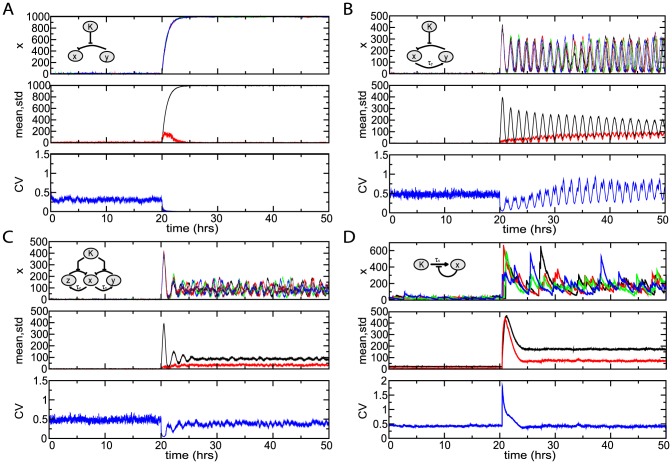
Stochastic model simulation results for various network architectures (with 1000 total NF-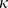
B molecules). The architectures analyzed are the NF-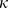
B network with no feedback loops (A), only I
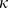
B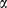-mediated negative feedback (B), the NF-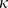
B network with both I
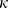
B
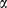
- and I
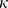
B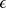-mediated negative feedback (C), and an alternative auto-repressive network (D). The top panel in each group shows four typical runs of stochastic simulations for each network, the middle panel shows the mean and standard deviation for 200 runs of each network, and the bottom panel shows the corresponding coefficient of variation. The input signal, K(t), is switched from 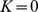 to 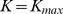 at 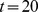 hrs. In A-C, the magnitude of external signal 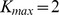, in D, 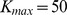.

The CV in late-phase nuclear NF-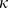
B levels is similar for extrinsic and intrinsic noise when the size of the system is reduced to 1000 NF-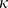
B molecules. Next, we investigated the behavior of the NF-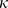
B signaling module in this regime where intrinsic noise levels become significant by analyzing stochastic simulations produced with a system with total NF-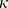
B levels set to 1000 molecules. We ran stochastic simulations of all three systems studied deterministically above: no-feedback, single negative feedback, and dual negative feedback (Figure 7). Note that ensemble-averaged time series agree with the deterministic simulations very well (Figure S6). In the case of no feedback (Figure 7*A*) there is a strong robust response to the incoming persistent signal as characterized by the low values of the coefficient of variation. However, as we have seen above in Figure 4*A*, the major flaw of this system is its slow response to the pulse-like signals. Next, we simulated the 9 biochemical reactions included in the I
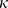
B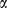-mediated single negative feedback loop (Figure 7*B*). In single runs the first peak in nuclear NF-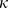
B levels appears to be very robust, as illustrated by Figure 7*B* *Top*. The CV is lowest (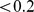) during the first peak in nuclear NF-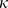
B indicating that this portion of the response is very robust. Subsequent peaks in this undamped system lead to higher CV (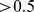) in the later portion of the response.

Next, we performed stochastic simulations of the 18 biochemical reactions included in the dual delayed feedback model (with both I
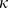
B
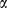
- and I
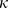
B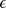-mediated feedback) (Figure 7*C*). In the dual feedback model, as in the single I
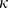
B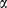-mediated feedback model, there is a very robust first peak. However, unlike the single I
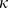
B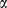-mediated feedback model, in the dual feedback system the noise levels remain at a low level (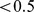) following the first peak in nuclear NF-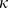
B (Figure 7*C* *Bottom*). Thus, the dual feedback architecture allows for lower noise levels also in the later portion of the response.

What is the underlying reason for the robustness of the initial response from this circuit? The main source of intrinsic noise lies in the transcription and translation of I
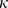
B isoforms, since they are transcribed from single genes. In contrast, fluctuations in protein degradation and transport processes are relatively small, because the copy numbers of the corresponding molecules are large. In the NF-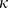
B network, the peak in nuclear NF-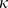
B levels that occurs following stimulation is produced via the degradation of I
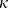
B proteins that bind and sequester NF-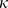
B in the cytoplasm. Thus, we argue that robustness of the initial response of the NF-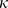
B circuit is explained by the fact that it uses the sequestering mechanism and does not rely on the protein production.

To test this hypothesis, we simulated the behavior of an alternative network that relies on transcription of auto-repressor, rather than the degradation of inhibitor proteins, for signaling (Figure 7*D*). This system can be modeled with two variables: 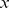, the number of repressor molecules, and 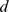, the binary state of the promoter (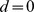 corresponds to the unbound promoter and 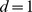 corresponds to bound promoter), and with four reactions (binding and unbinding of the repressor to the promoter, degradation of the repressor, and delayed synthesis of the repressor with rate 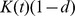 where 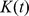 is the external signal (Tables S2, S3). The input signal activates the production of the auto-repressor which after a certain time delay binds to the promoter and terminates further synthesis. Deterministically, this circuit also provides a desired response to a persistent stimulation with a large well-defined first peak. However, stochastic simulations reveal significant differences in the noise performance of this design as compared with the NF-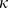
B circuit (note that the agreement between deterministic and stochastic simulations is less accurate in this case because of the strong promoter fluctuations (Figure S6 *D*). Activation of the auto-repressor network is much less robust than the activation of the NF-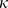
B network (cf. Figure 7*D* and Figures 7*B,C*). In fact, in the auto-repressor network, the coefficient of variation is highest (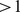) during the initial peak (Figure 7*D* *Bottom*). These results confirm our conjecture that the sequestering mechanism incorporated in the design of the NF-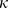
B network gives rise to a much more robust activation of NF-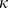
B than alternative networks that rely on transcription for activation and signaling. This finding is in accord with recent work [*31*] where the sequestering of Cdc20 protein was also implicated in the noise resistance of the spindle assembly checkpoint.

As we mentioned previously, recent computational work has suggested that persistent oscillations are present in wild-type cells with both I
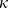
B
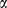
- and I
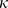
B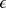-mediated feedback but stochastic variability leads to desynchronization among individual cells and therefore produces damped oscillations at the population level [13], [32]. Our computational results demonstrate that, although stochastic oscillations are still present in individual cells with both I
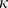
B
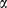
- and I
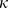
B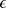-mediated feedback (Figure 7*C*), the oscillatory propensity can be greatly reduced by the second feedback loop in the wild-type NF-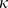
B signaling module. Further, stochastic simulations of the dual-feedback network reveal highly synchronized damped oscillations (Figure S7 *C*) with cellular variations due to intrinsic noise becoming significant only when the system size is drastically reduced (Figure 7*C*).

To show that our results are not limited to the conceptual NF-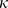
B model introduced above, we simulated the more detailed stochastic NF-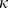
B model formulated in [32], which explicitly incorporates IKKK/IKK signaling cascade and NF-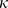
B shuttling between the nucleus and the cytoplasm (see Methods and Figure S8 *A*). One of the key assumptions made in the model [32] is that the strong stochasticity of the NF-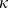
B response is caused by the slow and stochastic binding/dissociation of NF-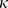
B to the corresponding promoters of I
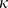
B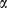, I
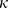
B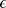, and A20 target genes. The slow rates chosen by the authors for these reactions lead to the high variability of oscillatory dynamics among cells (Figure S8 *B*). However, there is experimental evidence that the binding time of NF-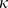
B may be significantly shorter, at least in certain types of cells. According to Fluorescence Recovery After Photobleaching (FRAP) measurements in HeLa cells [33], the typical time scale of NF-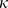
B binding to the target promoters is on the order of a second rather than minutes, suggesting more rapid equilibration between the NF-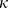
B-bound promoters and the pool of unbound nuclear NF-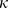
B molecules. We found that increasing the binding and dissociation rates by 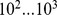 times profoundly changes the dynamics of the signaling system. NF-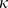
B trajectories become more regular, suggesting that the behavior of individual cells translates more directly into the behavior of the population (Figure S8 *C*). After adjusting the binding/dissociation rates along with a few other parameters (Table S5), the updated model recapitulated the population response to chronic TNF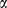 stimulation under various genetic conditions (WT, 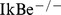, and 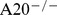) (Figure S9) in agreement with earlier experimental results [4], [21], [34].

**Figure 8:**
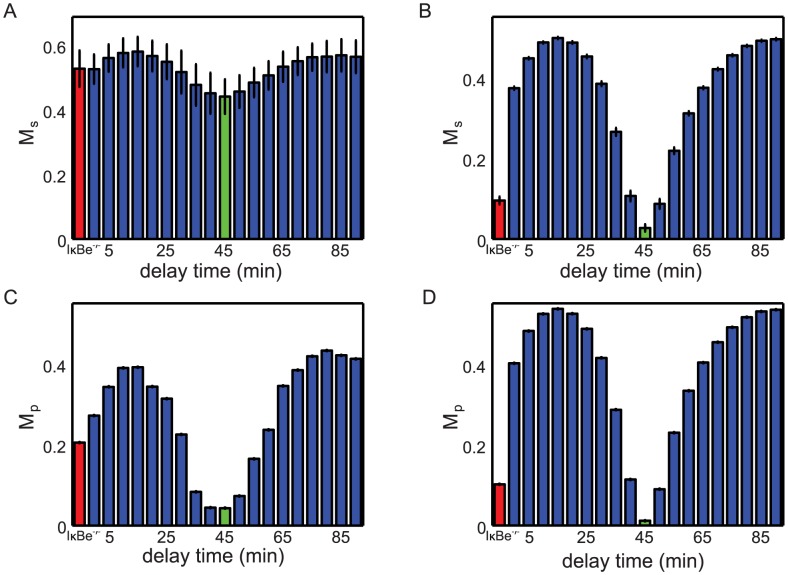
Effect of delay time on damped NF-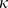
B oscillations in the detailed NF-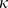
B model. Magnitudes of single-cell oscillations 5 hours after stimulation (A,B) and of population averaged oscillations (C,D) are shown for the 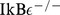 knockout (red bar) and in the WT for different time delays in the I
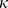
B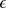 feedback loop (blue bars) with original (A,C) and adjusted (B,D) parameter values. The optimal time delay of 45 min is shown by the green bar. Each bar represents the average of nuclear NF-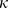
B variation for 500 single cell trajectories. Error bars represent 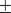 one standard deviation.

To quantify the magnitude of the late oscillatory NF-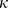
B response to a chronic TNF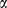 stimulation, we chose as a metric the average maximum peak-trough difference 5 hrs after initial stimulation. This quantity can be computed in two different ways. The mean single-cell variability can be characterized by the magnitude 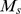 found by computing the maximum peak-trough differences for individual trajectories, and then averaging them over all trajectories:
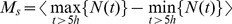
(8)The population-level variability can be characterized by the magnitude 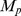 which is found by first computing an average trajectory and then computing its maximum peak-trough difference:
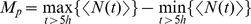
(9)If the stochasticity is small, these two measures are similar, however for strong stochasticity they may differ significantly. Using these metrics, we first confirmed that for the parameter values adopted by [32], the model shows significant single-cell oscillations both in the 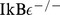 and in the WT, independently of the time delay in the I
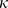
B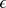 loop (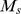, Figure 8*A*), but the population-averaged response shows significant oscillation dampening for the time delay around 45 min (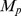, Figure 8*C*). However, for our re-parameterized model with fast binding/dissociation, the stochasticity of individual trajectories is small, and both metrics show similar trend: the amplitude of oscillations in the WT is strongly suppressed at the optimal time delay of 45 min both for the population average (Figure 8*B*) and the individual cells (Figure 8*D*), which falls within the margin of error of our experimental results (Figure 3*D*).

## Discussion

In this work we have developed a minimal model of the NF-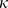
B signaling pathway that uses a small number of reactions (some of them compound) thus making it amenable to mathematical analysis. Previously, another simplified model of NF-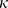
B signaling was developed in which a massive overshoot in I
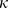
B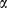 resulted in an effective slowing of signaling dynamics [35], and produced spiky oscillations that are not seen in physiological conditions. Our model, which utilizes an explicit time delay, recapitulates experimentally observed signaling behavior. It demonstrates that models with explicit time delays can be useful for investigating the mechanistic basis of the dynamic behavior of signaling pathways.

Using this model, we explored the potential role of NF-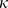
B oscillations which are observed in a variant of the NF-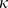
B signaling module with the secondary negative feedback loop involving I
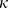
B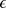, disabled. In particular, we addressed the question of whether the frequency of these oscillations contains information, as in neurons which sometimes encode information in the frequency of action potentials [36] and in the activation of the transcription factor NF-AT which is responsive to the number of 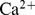 pulses [37]. By analyzing the oscillatory response of a system regulated solely by the I
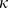
B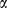-mediated negative feedback loop, we found that both the frequency and the decay rate of the oscillations produced by this system are highly dependent on the internal parameters of the circuit, but are not sensitive to changes in the input signal levels. This result suggests that the oscillatory frequency does not encode information about the stimulus. Hence, stimulus-specific gene expression is unlikely determined by stimulus-specific frequencies of NF-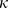
B oscillations. If there is a temporal code for stimulus-specific gene expression it is unlikely to involve frequency modulation, but may involve amplitude modulation over time.

When a second feedback regulator, I
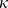
B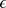, is added to the model, the oscillations caused by a persistent stimulation are significantly dampened, in agreement with our earlier findings [21]. By performing an optimization procedure, we determined that the specific experimentally observed parameter values for the synthesis delay and peak protein abundance of both I
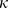
B isoforms correspond to maximal efficiency of damping. These findings suggest that the second feedback (I
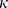
B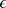) has evolved to produce damping of the oscillatory behavior of the first feedback (I
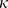
B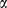). Furthermore, we demonstrated that this finding is not limited to our simple model, but can be expanded to more complex models. For example, in a recent model by [32] with fast binding/unbinding rates of NF-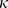
B the secondary I
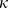
B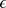 feedback leads to a reduction in NF-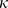
B oscillations in individual cells. However, cell-cell variability and extrinsic noise can further reduce NF-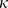
B oscillations on a population level.

From the evolutionary perspective, we have a peculiar situation in which a signaling module apparently first developed a negative feedback loop that made it prone to oscillations, and then added a secondary loop which mitigated these oscillations. This brings the question, if oscillatory responses are not beneficial to the cell, why has the primary negative feedback appeared in the system in the first place? By comparing transient response of several variants of signaling modules (0-, 1- and 2-feedback loop designs) in the presence of stochastic fluctuations we showed that the primary negative feedback loop involving the release of sequestered NF-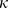
B proteins created a strong, rapid, and robust response to short pulses of active IKK signal. However, for longer signals a single-feedback-loop system exhibits a suboptimal “temporal dose response behavior” that leads to a quantized response to signals of different durations. In contrast, the dual feedback network generates response durations that are proportional to the stimulus input durations. Fine-tuning of the response duration may be reflective of a signaling code in which duration of NF-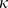
B activity may be a key determinant of stimulus-specific gene expression program.

Cytokines such as TNF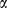 facilitate adaptive responses at the effector stages [38]. The evolution of cytokines is associated with the evolution of an adaptive immune system to allow for coordination of various cell types [39]. Unlike pathogen exposure, cytokines are produced during varying amounts of time thereby generating time-varying signals. Our analysis showed that the dual negative feedback module is more capable at distinguishing differences in the duration of incoming signals. This function is important for the transduction of cytokine signals, but not pathogen signals. Our BLASTP analysis indeed demonstrates that the evolution of the dual negative feedback system may correlate with the evolution of adaptive immunity.

## Methods

### Derivation of the deterministic model

Using mass action kinetics, the full set of reactions for the dual feedback loop NF-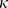
B system (Tables 2, 4) can be expressed by the following ODEs:
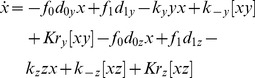
(10)

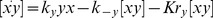
(11)

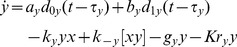
(12)

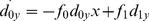
(13)

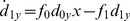
(14)

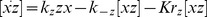
(15)

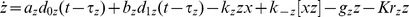
(16)

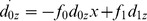
(17)

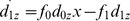
(18)


The total number of 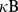 binding sites on each promoter is conserved:
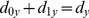
(19)

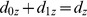
(20)We assume that the total amount of NF-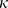
B in the cell 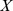 is conserved
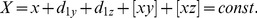
(21)


Since the number of binding sites available for NF-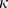
B protein is small, we can neglect the amount of NF-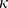
B bound to the I
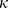
B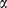 and I
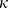
B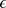 promoters, so
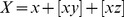
(22)Solving Eq. 22 for 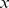 yields:
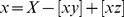
(23)DNA binding reactions are usually fast, so we can assume that they are at quasi-equilibrium at all times,
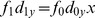
(24)

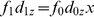
(25)Using Eqs. 19 and 20, substituting into Eqs. 24 and 25, and solving for 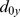, 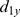, 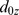, 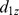 yields:
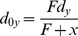
(26)

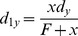
(27)

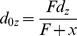
(28)

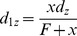
(29)where 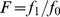.

We also assume quasi-equilibrium for I
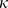
B NF-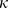
B binding reactions,
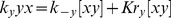
(30)

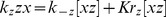
(31)Substituting 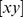 and 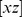 from Eqs. 30 and 31 into Eq. 23 yields:
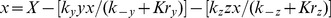
(32)Now we can solve Eq. 32 for 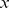


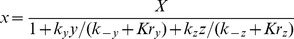
(33)and substitute it in Eqs. 12 and 16. These equations contain both fast and slow terms. However, it is easy to see that rate equations for variables 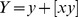 and 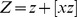 contain only slow terms:
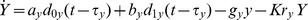
(34)

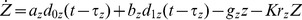
(35)

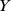
 and 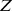 can in turn be expressed via 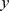 and 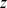 by:
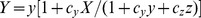
(36)

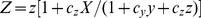
(37)where 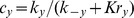 and 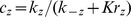. Equations 34–35 combined with definitions Eqs. 26–29, 33, 36, and 37 represent a closed system of two delay-differential equations 2, 3 for the dual-feedback NF-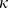
B module. Setting 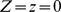 in these equations leaves us with a single delay-differential equation for the single feedback loop system Eq. 1.

### Details of the linear stability analysis

The fixed point 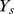 of Eq. (4) is given by the algebraic equation (6). Unfortunately, Eq. (6) does not permit finding 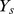 in explicit form. However, this calculation can be significantly simplified if the total number of NF-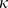
B proteins is large, so 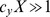, then 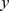 can be neglected as compared with total 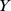. Then 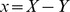, and 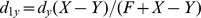, and expression (5) for 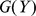 simplifies:
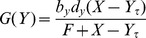
(38)Now the stationary level of 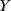 can be obtained explicitly
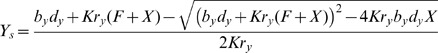
(39)


The stability of this stationary solution is determined by the linearized equation (4) for a small perturbation 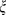 near 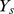,
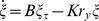
(40)where 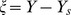, subscript 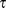 again indicates the delayed value of 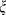 taken at time 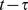, and 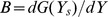. Using formula (38) we obtain
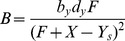
(41)where 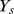 is given by Eq. (39). The eigenvalue 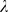 of the linearized equation (40) is found by substituting 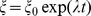, yielding the transcendental equation
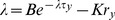
(42)whose solution is given by Eq. (7).

### Stochastic model formulation

For the analysis of a full NF-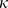
B system, we adopted the basic structure of the NF-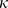
B model formulated in [32] which in turn was based on the population-level model first proposed in [4]. The structure of the model is shown in Figure S8 *A*. In resting cells, NF-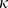
B is sequestered in the cytoplasm by I
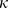
B proteins. In response to TNF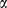 stimulation, IKKK protein becomes active, and activates IKK kinase. IKK phosphorylates I
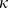
B proteins targeting them for degradation. Upon degradation of I
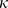
B proteins, NF-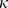
B moves into the nucleus and activates hundreds of target genes. In the model, we focus on the dynamics of three genes associated with the negative feedback of the system. Following NF-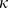
B activation, synthesized A20 proteins attenuate TNF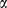 signal by repressing IKKK and IKK transitions into their active states. NF-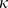
B also binds I
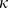
B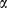 and I
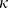
B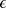 protein promoters, which following translation in the cytoplasm, translocate back into the nucleus and bind free NF-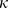
B sequestering it out of the nucleus. In addition, I
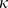
B proteins are directly responsible for NF-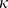
B dissociation from the DNA.

The biological processes in the model were interpreted through stochastic and deterministic representations similar to [32]. Nuclear transport, complex formation, synthesis, transcription, and translation were described through a set of ordinary differential equations (Text S1). Regulation of gene activity through NF-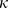
B binding and dissociation from DNA was modeled using stochastic representation. The time-evolution of the system was accomplished through a hybrid simulation algorithm that uses Gillespie algorithm [29] to evaluate the state of stochastic processes and an ODE solver to compute the state of deterministic processes.

### Details of the BLASTP search for I
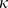
B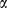 and I
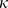
B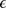 homologs

We performed two BLASTP searches (using default parameters) to search for I
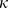
B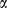 and I
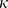
B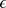 homologs. The mouse I
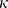
B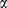 protein sequence (gi28386026) was used as the query for the first search. The mouse I
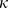
B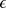 protein sequence (gi2739158) was used as the query for the second search. We used an E-value of 1e-25 as a cutoff for both searches. Homologs for I
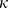
B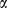 were found in the organisms listed in Table S6, and homologs for I
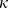
B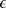 were found in the organisms listed in Table S7.

Note that we selected only unique homologs for both I
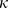
B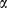 and I
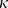
B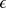 in all vertebrates. We did not find unique I
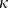
B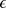 homologue for several vertebrates. We expect that this is due to the fact that complete genomes are not currently available for these organisms. Table S8 lists the genome status (as of 6/1/11) of all organisms for which I
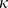
B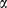 or I
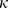
B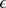 homologs were found (http://www.ncbi.nlm.nih.gov/genomes/leuks.cgi).

### Cell culture experiments

Immortalized murine embryonic fibroblasts [4] were chronically stimulated with 10 ng/mL TNF (Roche) and I
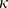
B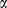 and I
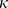
B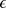 mRNA and protein levels were monitored by RNase Protection Assay (RPA) and Western Blot, respectively, as previously described [21]. RPA results for each time course were quantitated using ImageQuant software (GE Healthcare) and used to determine the time of half-maximal inducibility between basal and peak mRNA levels for I
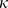
B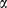 and I
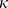
B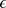 (Figure S2 *A,B*). Western Blot results were also quantitated with ImageQuant software and used to determine the time point of peak expression. The basal abundances of I
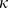
B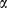 and I
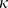
B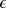 protein were determined via comparison to a standard curve of recombinant I
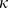
B protein (R Tsu, JD Kearns, C Lynch, D Vu, K Ngo, S Basak, G Ghosh, A Hoffmann *in preparation*). The peak abundances of I
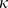
B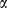 and I
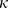
B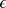 were determined via multiplication of the basal value by the fold inducibility at the peak time point (Figure S2 *C,D*). Experimental levels of nuclear NF-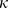
B in cells with only the I
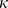
B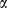-mediated negative feedback loop intact and in wild-type cells containing both I
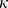
B
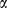
- and I
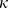
B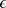-mediated negative feedback were determined by EMSAs in [4].

## Supporting Information

Figure S1
**Oscillations produced by the I** 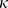 **B** 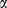 **-mediated negative feedback system.** (A) 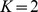 for 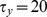 min, 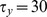 min, and 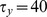 min and with (B) 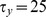 min for 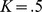, 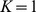, and 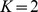.(EPS)

Figure S2
**Representative experimental data for I** 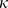 **B** 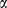 **and I** 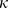 **B** 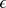 **synthesis delays and feedback strengths.** (A) mRNA synthesis for I
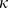
B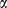 and I
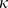
B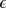 were measured by RNase Protection Assay in wild-type immortalized MEF cells in response to 10 ng/mL TNF chronic stimulation. (B) The RPA results were quantitated to determine the intensity of each band in the gel (ImageQuant, GE Healthcare). The highest intensity band in each set was set to 100% Activation and the other bands were normalized accordingly. The delay time to reach half maximal synthesis was calculated as the time at which the activation curve crossed the 50% level. A set of N = 10 replicate experiments were performed to calculate the global average. (C) The protein abundances for I
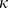
B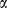 and I
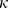
B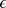 at their respective activation peaks in wild-type immortalized MEF cells chronically stimulated with 10 ng/mL TNF were measured by Western Blot analysis (I
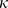
B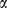 at 1 h and I
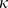
B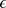 at 6 h) . Fold induction vs. basal state are shown below each gel and were calculated by quantitation of the band intensities and normalization to the 0 h band. (D) Bar plot of the average protein abundances from multiple Western Blot experiments for peak levels of I
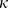
B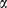 (N = 7) and I
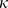
B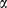 (N = 5). The basal state abundances were measured by comparison to a standard curve of recombinant I
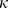
B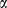 or I
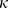
B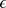 protein (JD Kearns, S Basak, C Lynch, A Hoffmann *in preparation*). Peak abundances were calculated by multiplying the quantitated fold induction (as in C) by the basal abundance. Error bars on the peak bars represent one standard deviation.(EPS)

Figure S3
**The ratio of peak I** 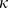 **B** 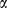 **protein levels to peak I** 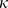 **B** 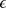 **protein levels (** 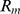 **) versus** 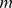 **.** 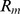 was determined for several values of 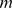 in the model simulations to determine the value of 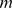 corresponding to 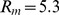 (The experimentally measured value for the ratio is 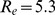). This value of 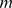 (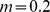) was used to plot the point in Figure 3D which indicates the experimental values of 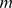 and 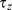.(EPS)

Figure S4
**Response of a no-feedback system with constitutive I** 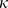 **B** 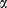 **synthesis increased from** 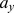 **= 0.00185 nM/min to** 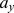 **= 0.3 nM/min.** The time series of 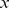 is shown for 15 min (red), 30 min (orange), 45 min (green), and persistent (blue) stimulation.(EPS)

Figure S5
**Coefficient of variation (CV) of nuclear NF-** 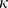 **B levels.** Comparison of the CV for peak and late-phase nuclear NF-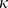
B levels due to extrinsic variability in total NF-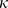
B (A,C) and IKK (B,D) for the single feedback and dual feedback network.(EPS)

Figure S6 **Comparison of ensemble-averaged runs of stochastic simulations with deterministic simulations for four different circuits.** (A) no-feedback model, (B) single negative feedback model, (C) dual feedback system, (D) and auto-repressor system. Lines - deterministic simulations, symbols - stochastic simulations averaged over 200 runs.
(EPS)

Figure S7
**Stochastic simulation results with 100,000 total NF-** 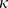 **B molecules.** Four NF-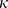
B networks were considered: no feedback loops (A), only I
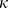
B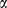-mediated negative feedback (B), the NF-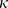
B network with both I
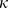
B
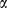
- and I
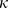
B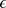-mediated negative feedback (C), and an alternative auto-repressive network (D). The top panel in each group shows four typical runs of stochastic simulations for each network, the middle panel shows the mean and standard deviation for 200 runs of each network, and the bottom panel shows the corresponding coefficient of variation. The input signal, K(t), is switched from 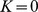 to 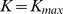 at 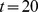 hrs. In A–C, the magnitude of external signal 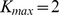, in D, 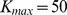.(EPS)

Figure S8
**Oscillatory behavior from NF-** 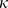 **B signaling system.** (A) Diagram of the NF-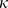
B signaling network model adopted from Paszek *et. al* (2010). In resting cells, NF-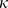
B is sequestered in the cytoplasm by I
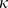
B proteins. In response to TNF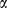 stimulation, IKKK protein becomes active, activating IKK kinase. In turn, IKK phosphorilates I
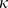
B proteins targeting them for degradation. Upon degradation of I
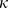
B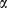, NF-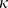
B moves into the nucleus and activates hundreds of target genes. In the model, we describe the dynamics of three genes associated with the negative feedback of the system. Following NF-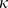
B activation, synthesized A20 proteins attenuate TNF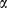 signal by repressing IKKK and IKK transitions into their active states. NF-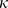
B also binds I
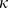
B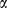 and I
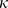
B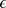 protein promoters, which following translation in the cytoplasm, translocate back into the nucleus and bind up free NF-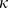
B sequestering it out of the nucleus. In addition, I
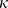
B proteins are directly responsible for NF-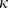
B dissociation from the DNA. (B) Nuclear NF-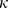
B levels in response to persistent stimulation as a function of time computed using Paszek *et. al* (2010) wildtype model. (C) Nuclear NF-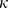
B levels in response to persistent stimulation as a function of time computed using our re-parameterized model. Black curve represents an average of 500 cell trajectories.(EPS)

Figure S9
**Nuclear NF-** 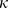 **B response in A20 and I** 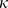 **B** 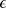 **knockout models to chronic TNF** 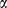 **stimulation.** The results for the updated model (C,D) shows close similarity, in NF-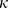
B population dynamics, to the results obtained using Paszek *et. al* (2010) wildtype model (A,B) for both I
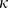
B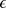 knockout (A,C) and A20 knockout (B,D) models. Black trajectories represent the average of 500 cell trajectories.(EPS)

Table S1 **Model parameter values.** Model schematic shown in Figure 3A.
(PDF)

Table S2 **Auto-repressor network reactions.**
(PDF)

Table S3 **Auto-repressor network parameter values.**
(PDF)

Table S4 **Stochastic model variables.** Model schematic shown in Figure S8A.
(PDF)

Table S5 **Stochastic model parameter values.** Model schematic shown in Figure S8A.
(PDF)

Table S6
**Organisms with homologs for I** 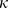 **B** 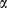 **.** BLASTP analysis results.(PDF)

Table S7
**Organisms with homologs for I** 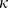 **B** 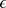 **.** BLASTP analysis results.(PDF)

Table S8 **Genome status of organisms in Tables S6 and S7.**
(PDF)

Text S1 **Additional model details.** Extrinsic noise in dual negative feedback loop system and details of the full stochastic model.
(PDF)
