# Supplementary material for: Dual Delayed Feedback Provides Sensitivity and Robustness to the NF-κB Signaling Module
Source: PLoS Comput Biol. 2013 Jun 27;9(6):e1003112. doi: 10.1371/journal.pcbi.1003112 (PMC3694842; doi:10.1371/journal.pcbi.1003112)
Supplement: Table S1 — Model parameter values. Model schematic shown in Figure 3A. (PDF) [file pcbi.1003112.s010.pdf]

Table S1. Model parameter values

	Parameter Description	Model Value**	Ref. Value	Ref.
a_y	IκBα constitutive synthesis*	0.00185 nM/min	0.0185 nM/min	(Kearns *et al*, 2006)
b_y	IκBα inducible synthesis*	5.0 nM/min	138.6 nM/min	(Kearns *et al*, 2006)
d_y	IκBα promoter: number κB sites	1.0		
f_0	NF-κB binding IκB promoter	10.0 /(nMmin)		
f_1	NF-κB unbinding IκB promoter	200.0/min		
τ_y	Delay in IκBα synthesis	25.0 min	25.8 min	this work
k_y	IκBα binding with NF-κB	0.1 /(nMmin)	0.03 /(nMmin)	(Kearns *et al*, 2006)
k_{-y}	IκBα dissociates from NF-κB	0.0006/min	0.00006/min	(Kearns *et al*, 2006)
X	Total NF-κB	100 nM	100 nM	(Hoffmann *et al*, 2002)
g_y	Constitutive IκBα degradation	0.012/min	0.12/min	(Kearns *et al*, 2006)
r_y	Degradation of IκBα by IKK	0.01/min	0.018/min	(Kearns *et al*, 2006)
a_z	IκBϵ constitutive synthesis*	0.00046 nM/min	0.0046 nM/min	(Kearns *et al*, 2006)
b_z	IκBϵ inducible synthesis*	5.0 nM/min	18.0 nM/min	(Kearns *et al*, 2006)
d_z	IκBϵ promoter: number κB sites	1.0		
τ_z	Delay in IκBϵ synthesis	72.0 min	59.4 min	this work
k_z	IκBϵ binding with NF-κB	0.1 /(nMmin)	0.03 /(nMmin)	(Kearns *et al*, 2006)
k_{-z}	IκBϵ dissociates from NF-κB	0.0006/min	0.00006/min	(Kearns *et al*, 2006)
g_z	Constitutive IκBϵ degradation	0.018/min	0.18/min	(Kearns *et al*, 2006)
r_z	Degradation of IκBϵ by IKK	0.01/min	0.018/min	(Kearns *et al*, 2006)

*per 1 κB site

***1 nM = 1000 molecules for a cell volume that is on the order of 1 pico liter
