# Supplementary material for: Dual Delayed Feedback Provides Sensitivity and Robustness to the NF-κB Signaling Module
Source: PLoS Comput Biol. 2013 Jun 27;9(6):e1003112. doi: 10.1371/journal.pcbi.1003112 (PMC3694842; doi:10.1371/journal.pcbi.1003112)
Supplement: Table S2 — Auto-repressor network reactions. (PDF) [file pcbi.1003112.s011.pdf]

**Table S2. Auto-repressor network reactions**

| Reaction | Rate | Description |
| --- | --- | --- |
| $d_0 + x \leftrightarrow d_1$ | $k_y$, $k_{-y}$ | binding and unbinding of the repressor to the promoter |
| $x \rightarrow \phi$ | $g_y$ | degradation of the repressor |
| $d_0 \rightarrow d_0 + x$ | $a_y(1 + K)$ | synthesis of the repressor (delayed reaction) |
