# Supplementary material for: Dual Delayed Feedback Provides Sensitivity and Robustness to the NF-κB Signaling Module
Source: PLoS Comput Biol. 2013 Jun 27;9(6):e1003112. doi: 10.1371/journal.pcbi.1003112 (PMC3694842; doi:10.1371/journal.pcbi.1003112)
Supplement: Table S3 — Auto-repressor network parameter values. (PDF) [file pcbi.1003112.s012.pdf]

**Table S3. Auto-repressor network parameter values**

| | **Parameter Description** | **Parameter Value** |
|---|---|---|
| $a_y$ | repressor synthesis rate | 1.0 nM/min |
| $g_y$ | repressor degradation rate | 0.012/min |
| $k_y$ | binding of repressor to promoter | 0.1 nM/min |
| $k_{-y}$ | unbinding of repressor from promoter | 0.06/min |
| $\tau_z$ | delay in synthesis of repressor | 25.0 min |
