# Supplementary material for: Dual Delayed Feedback Provides Sensitivity and Robustness to the NF-κB Signaling Module
Source: PLoS Comput Biol. 2013 Jun 27;9(6):e1003112. doi: 10.1371/journal.pcbi.1003112 (PMC3694842; doi:10.1371/journal.pcbi.1003112)
Supplement: Table S4 — Stochastic model variables. Model schematic shown in Figure S8A. (PDF) [file pcbi.1003112.s013.pdf]

Table S4. Stochastic model variables

**Variable**	**Description**
IKKKa	Active form of *IKKK* kinase
IKKa	Active form of *IKK* kinase
IKKi	Inactive form of *IKK* kinase
IKKn	Neutral form of *IKK* kinase
$G_{I\kappa B\alpha}$	Discrete state of IκBα gene promoter
$G_{I\kappa B\epsilon}$	Discrete state of IκBϵ gene promoter
G_{A20}	Discrete state of A20 gene promoter
$NF\text{-}\kappa B$	Free cytoplasmic NF-κB
$nNF\text{-}\kappa B$	Free nuclear NF-κB
$I\kappa B\alpha$	Free cytoplasmic IκBα
$I\kappa B\epsilon$	Free cytoplasmic IκBϵ
$nI\kappa B\alpha$	Free nuclear IκBα
$nI\kappa B\epsilon$	Free nuclear IκBϵ
A20	Cytoplasmic A20 protein
$tI\kappa B\alpha$	IκBα mRNA transcript
$tI\kappa B\epsilon$	IκBϵ mRNA transcript
tA20	A20 mRNA transcript
$pI\kappa B\alpha$	Phosphorilated IκBα
$pI\kappa B\epsilon$	Phosphorilated IκBϵ
$I\kappa B\alpha\_NF\text{-}\kappa B$	Cytoplasmic NF-κB and IκBα complex
$I\kappa B\epsilon\_NF\text{-}\kappa B$	Cytoplasmic NF-κB and IκBϵ complex
$nI\kappa B\alpha\_NF\text{-}\kappa B$	Nuclear NF-κB and IκBα complex
$nI\kappa B\epsilon\_NF\text{-}\kappa B$	Nuclear NF-κB and IκBϵ complex
$pI\kappa B\alpha\_NF\text{-}\kappa B$	Cytoplasmic NF-κB and phospho-IκBα complex
$pI\kappa B\epsilon\_NF\text{-}\kappa B$	Cytoplasmic NF-κB and phospho-IκBϵ complex
