# Supplementary material for: Dual Delayed Feedback Provides Sensitivity and Robustness to the NF-κB Signaling Module
Source: PLoS Comput Biol. 2013 Jun 27;9(6):e1003112. doi: 10.1371/journal.pcbi.1003112 (PMC3694842; doi:10.1371/journal.pcbi.1003112)
Supplement: Table S5 — Stochastic model parameter values. Model schematic shown in Figure S8A. (PDF) [file pcbi.1003112.s014.pdf]

Table S5. Stochastic model parameter values

	Value	Unit	Parameter Description	Reference
Protein synthesis and degradation				
c1,a	0.1	s^{-1}	IκBα inducible mRNA synthesis	(Paszek *et al*, 2010)
**c1e**	0.07	s^{-1}	IκBϵ inducible mRNA synthesis	Fitted
c2,a,e	0.5	s^{-1}	Protein translation	(Paszek *et al*, 2010)
c3,a,e	0.00048	s^{-1}	mRNA degradation	(Paszek *et al*, 2010)
c4a,e	0.0001	s^{-1}	Free IκBα degradation	(Paszek *et al*, 2010)
c4	0.0045	s^{-1}	A20 degradation	(Werner *et al*, 2008)
Complex parameters				
c5a,e	0.00001	s^{-1}	Degradation of complexed IκB	(Paszek *et al*, 2010)
**ka1a,e**	3.33^{-7}	s^{-1}	NF-κB-IκB association	Fitted
kd1a,e	$5{\cdot}10^{-4}$	s^{-1}	IκB_NF-κB dissociation	(Paszek *et al*, 2010)
kc1a,e	$5{\cdot}10^{-7}$	s^{-1}	IKKa phosphorylation of free IκB	(Paszek *et al*, 2010)
kc2a,e	$2{\cdot}10^{-6}$	s^{-1}	IKKa phosphorylation of $I\kappa B\_NF\text{-}\kappa B$	(Paszek *et al*, 2010)
kt1a,e	0.1	s^{-1}	Degradation of free $pI\kappa B$	(Paszek *et al*, 2010)
kt2a,e	0.1	s^{-1}	Degradation of complexed $pI\kappa B$	(Paszek *et al*, 2010)
ke2a,e	0.05	s^{-1}	IκB_NF-κB nuclear export	(Paszek *et al*, 2010)
**ki3a,e**	0.0005	s^{-1}	IκB nuclear import	Fitted
**ke3a,e**	0.00125	s^{-1}	IκB nuclear export	Fitted
IKKK and IKK activation				
kr	0.025	s^{-1}	IKKKn activation by TNFα	(Paszek *et al*, 2010)
kri	0.0003	s^{-1}	IKKKa $\rightarrow$ IKKKn	(Paszek *et al*, 2010)
kp	$1.75{\cdot}10^{-4}$	s^{-1}	IKKii $\rightarrow$ IKKn	(Paszek *et al*, 2010)
ka	$7{\cdot}10^{-7}$	s^{-1}	IKKn $\rightarrow$ IKKa	(Paszek *et al*, 2010)
ki	0.005	s^{-1}	IKKa $\rightarrow$ IKKi	(Paszek *et al*, 2010)
kii	0.0015	s^{-1}	IKKi $\rightarrow$ IKKii	(Paszek *et al*, 2010)
kaA20	$3{\cdot}10^{-7}$	s^{-1}	IKKKn inactivation by A20	(Paszek *et al*, 2010)
kbA20	$6{\cdot}10^{3}$	s^{-1}	A20 inhibition of IKKii $\rightarrow$ IKKn	(Paszek *et al*, 2010)
Other parameters				
ki1	0.005	s^{-1}	NF-κB nuclear import	(Paszek *et al*, 2010)
**q1**	$1.5{\cdot}10^{-4}$	s^{-1}	nfkb DNA binding	(Bosisio *et al*, 2006)
**q2a,e**	10^{-3}	s^{-1}	IκB-mediated NF-κB dissociation from DNA	(Bosisio *et al*, 2006)
-	60,000		Total NF-κB	(Paszek *et al*, 2010)
$K_N N$	10,000		Total level of IKKK	(Paszek *et al*, 2010)
K_N	200,000		Total level of IKK	(Paszek *et al*, 2010)
-	60,000		Total NF-κB	(Paszek *et al*, 2010)
T_D	45	min	Delay in IκBϵ tanscription	Fitted
tv	$2{\cdot}10^{-}12$	l	Total cell volume	(Paszek *et al*, 2010)
kv	5		Cytoplasm:Nucleus ratio	(Paszek *et al*, 2010)
TR	1		TNFα input (10ng/ml)	(Paszek *et al*, 2010)
