# Supplementary material for: Dual Delayed Feedback Provides Sensitivity and Robustness to the NF-κB Signaling Module
Source: PLoS Comput Biol. 2013 Jun 27;9(6):e1003112. doi: 10.1371/journal.pcbi.1003112 (PMC3694842; doi:10.1371/journal.pcbi.1003112)
Supplement: Table S6 — Organisms with homologs for I B . BLASTP analysis results. (PDF) [file pcbi.1003112.s015.pdf]

Table S6. Organisms with homologs for IκBα

Organism	Common name	NCBI gi number	E-value
Mus musculus	mouse	28386026	0
Rattus norvegicus	rat	160333919	0
Sus scrofa	pig	52346212	9.00E-169
Canis lupus familiaris	dog	73963074	1.00E-168
Bos taurus	cow	114052817	3.00E-168
Oryctolagus cuniculus	rabbit	291403740	7.00E-168
Ovis aries	sheep	261244990	1.00E-167
Pongo abelii	orangutan	297694934	2.00E-167
Macaca mulata	rhesus monkey	109083336	6.00E-167
Macaca fascicularis	macaque	90080313	6.00E-167
Homo sapiens	human	10092619	1.00E-166
Ailuropoda melanoleuca	giant panda	301774897	2.00E-166
Callithrix jacchus	marmoset	296214818	5.00E-165
Nomascus leucogenys	gibbon	332229160	9.00E-150
Monodelphis domestica	opossum	126283568	2.00E-147
Ornithorhynchus anatinus	platypus	149548454	8.00E-138
Gorilla gorilla	gorilla	120974064	2.00E-126
Equus caballus	horse	194207294	7.00E-126
Pan paniscus	bonobo	121483818	2.00E-125
Taeniopygia guttata	songbird	224051436	6.00E-118
Pan troglodytes	chimp	124111100	4.00E-117
Anolis carolinensis	anole	327263645	9.00E-117
Gallus gallus	chicken	126723285	2.00E-116
Xenopus tropicalis	western clawed frog	52345606	3.00E-95
Meleagris gallopavo	turkey	326920741	8.00E-95
Xenopus laevis	African clawed frog	148230967	5.00E-94
Salmo salar	salmon	209737318	6.00E-80
Danio rerio	zebrafish	37725732	3.00E-72
Gadus morhua	Atlantic cod	296044660	2.00E-68
Tetraodon nigroviridis	pufferfish	47209080	3.00E-63
Lemur catta	lemur	122938174	1.00E-44
Apis mellifera	honey bee	110756132	7.00E-34
Biomphalaria glabrata	snail	119393872	2.00E-31
Nasonia vitripennis	wasp	156543541	8.00E-30
Lutzomyia longipalpis	sandfly	149728129	2.00E-29
Drosophila yakuba	fly	195475366	7.00E-29
Aedes aegypti	mosquito	157108525	9.00E-29
Pediculus humanus corporis	human body louse	212512255	1.00E-28
Drosophila willistoni	fly	195433326	1.00E-28
Drosophila erecta	fly	194857830	2.00E-28
Drosophila grimshawi	fly	195034537	2.00E-28
Drosophila melanogaster	fruit fly	17136840	2.00E-28
Drosophila virilis	fly	195386110	2.00E-28
Drosophila mojavensis	fly	195114618	4.00E-28
Drosophila persimilis	fly	195160098	6.00E-28
Drosophila pseudoobscura	fly	198475712	7.00E-28
Drosophila ananassae	fly	194759838	7.00E-28
Camponotus floridanus	ant	307177756	1.00E-27
Nematostella vectensis	sea anemone	156079906	2.00E-27
Harpegnathos saltator	ant	307194384	3.00E-27
Strongylocentrotus purpuratus	sea urchin	72004276	3.00E-27
Ciona intestinalis	sea squirt	118343834	3.00E-27
Daphnia pulex	flea	321478250	4.00E-27
Tribolium castaneum	beetle	91093127	1.00E-26
Solenopsis invicta	ant	322786068	5.00E-26
Anopheles gambiae str. PEST	mosquito	118789617	6.00E-26
