# Supplementary material for: Dual Delayed Feedback Provides Sensitivity and Robustness to the NF-κB Signaling Module
Source: PLoS Comput Biol. 2013 Jun 27;9(6):e1003112. doi: 10.1371/journal.pcbi.1003112 (PMC3694842; doi:10.1371/journal.pcbi.1003112)
Supplement: Table S7 — Organisms with homologs for I B . BLASTP analysis results. (PDF) [file pcbi.1003112.s016.pdf]

Table S7. Organisms with homologs for IκBϵ

Organism	Common name	NCBI gi number	E-value
Mus musculus	mouse	2739158	0
Rattus norvegicus	rat	40018590	0
Bos taurus	cow	195539539	7.00E-161
Ailuropoda melanoleuca	panda	301757312	3.00E-159
Callithrix jaccus	marmoset	296198283	5.00E-156
Nomascus leucogenys	gibbon	332234141	6.00E-155
Pan troglodytes	chimp	114607603	1.00E-153
Homo sapiens	human	20530139	2.00E-153
Macaca mulatta	rhesus monkey	297290951	9.00E-150
Monodelphis domestica	opossum	126310114	5.00E-129
Canis familiaris	dog	73973023	2.00E-89
Anolis carolinensis	anole	327262290	1.00E-82
Xenopus tropicalis	western clawed frog	187608250	7.00E-81
Xenopus laevis	African clawed frog	147907302	5.00E-74
Danio rerio	zebrafish	121583661	1.00E-61
Salmo salar	salmon	213514346	3.00E-61
Tetraodon nigroviridis	pufferfish	47224342	6.00E-53
Gallus gallus	chicken	118088055	3.00E-38
Ornithorhynchus anatinus	platypus	149542664	1.00E-35
Equus caballus	horse	194205729	2.00E-29

The IκBϵ sequences of above organisms are different from the sequences that were found with the IκBα search.
