# Supplementary material for: Dual Delayed Feedback Provides Sensitivity and Robustness to the NF-κB Signaling Module
Source: PLoS Comput Biol. 2013 Jun 27;9(6):e1003112. doi: 10.1371/journal.pcbi.1003112 (PMC3694842; doi:10.1371/journal.pcbi.1003112)
Supplement: Table S8 — Genome status of organisms in Tables S6 and S7. (PDF) [file pcbi.1003112.s017.pdf]

Table S8. Genome status of organisms in Tables S6 and S7

Organism	Common name	Genome status
Aedes aegypti	mosquito	Assembly
Ailuropoda melanoleuca	giant panda	Assembly
Anolis carolinensis	anole	Assembly
Anopheles gambiae str. PEST	mosquito	Assembly
Apis mellifera	honey bee	Assembly
Biomphalaria glabrata	snail	In Progress
Bos taurus	cow	Assembly
Callithrix jacchus	marmoset	Assembly
Camponotus floridanus	ant	Assembly
Canis lupus familiaris	dog	Assembly
Ciona intestinalis	sea squirt	Assembly
Danio rerio	zebrafish	Assembly
Daphnia pulex	flea	Assembly
Drosophila ananassae	fly	Assembly
Drosophila erecta	fly	Assembly
Drosophila grimshawi	fly	Assembly
Drosophila melanogaster	fruit fly	Assembly
Drosophila mojavensis	fly	Assembly
Drosophila persimilis	fly	Assembly
Drosophila pseudoobscura	fly	Assembly
Drosophila virilis	fly	Complete
Drosophila willistoni	fly	Assembly
Drosophila yakuba	fly	Assembly
Equus caballus	horse	Assembly
Gadus morhua	Atlantic cod	In Progress
Gallus gallus	chicken	Assembly
Gorilla gorilla	gorilla	Assembly
Harpegnathos saltator	ant	Assembly
Homo sapiens	human	Complete
Lemur catta	lemur	In Progress
Lutzomyia longipalpis	sandfly	In Progress
Macaca fascicularis	macaque	In Progress
Macaca mulata	rhesus monkey	Assembly
Meleagris gallopavo	turkey	Assembly
Monodelphis domestica	opossum	Assembly
Mus musculus	mouse	Complete
Nasonia vitripennis	wasp	Assembly
Nematostella vectensis	sea anemone	Assembly
Nomascus leucogenys	gibbon	Assembly
Ornithorhynchus anatinus	platypus	Assembly
Oryctolagus cuniculus	rabbit	Assembly
Ovis aries	sheep	Assembly
Pan paniscus	bonobo	In Progress
Pan troglodytes	chimp	Complete
Pediculus humanus corporis	human body louse	Assembly
Pongo abelii	orangutan	Assembly
Rattus norvegicus	rat	Assembly
Salmo salar	salmon	Not Found
Solenopsis invicta	ant	Assembly
Strongylocentrotus purpuratus	sea urchin	Assembly
Sus scrofa	pig	Assembly
Taeniopygia guttata	songbird	Assembly
Tetraodon nigroviridis	pufferfish	Assembly
Tribolium castaneum	beetle	Assembly
Xenopus laevis	African clawed frog	Not Found
Xenopus tropicalis	western clawed frog	Assembly
