# Supplementary material for: Dual Delayed Feedback Provides Sensitivity and Robustness to the NF-κB Signaling Module
Source: PLoS Comput Biol. 2013 Jun 27;9(6):e1003112. doi: 10.1371/journal.pcbi.1003112 (PMC3694842; doi:10.1371/journal.pcbi.1003112)
Supplement: Text S1 — Additional model details. Extrinsic noise in dual negative feedback loop system and details of the full stochastic model. (PDF) [file pcbi.1003112.s018.pdf]

Text S1

Extrinsic noise in dual negative feedback loop system

We find that the CV in peak nuclear NF-κB increases linearly with extrinsic variation in total NF-κB and with extrinsic variation in IKK with identical CV values for both the single and dual feedback models (Figure S5*A,B*). In contrast, the CV in late-phase (asymptotic) NF-κB levels are significantly lower in the dual feedback system than in the single feedback system. We varied the magnitude of extrinsic noise by changing the spread of parameters (total NF-κB and IKK) from 0% to 50%. The CV in late-phase NF-κB for the dual feedback system increases linearly from 0 to approximately 1 as the range of total NF-κB (Figure S5*C*) and IKK (Figure S5*D*) is increased to ±50%, while the CV in late-phase NF-κB for the single feedback system increases from approximately 1.6 to 1.9 (Figure S5*C,D*). Thus, in the presence of extrinsic variations in IKK and total NF-κB, the dual feedback system allows for a late-phase response which is more robust than the response produced by the single feedback system.

Details of the full stochastic model

For the analysis of a full NF-κB system, we adopted the basic structure of the NF-κB model formulated in Paszek et. al., 2010. The structure of the model is shown in Figure S8*A*. The biological processes in the model were interpreted through stochastic and deterministic representations. Nuclear transport, complex formation, synthesis, transcription, and translation were described through a set of ordinary differential equations (ODEs). Regulation of gene activity through NF-κB binding and dissociation from DNA was modeled using stochastic representation. The time-evolution of the system was accomplished through a hybrid simulation algorithm that uses Gillespie algorithm (Gillespie, 1977) to evaluate the state of stochastic processes and an ODE solver to compute the state of deterministic processes.

ODEs

See Tables S4 and S5 for variable and parameter descriptions respectively.

$$\begin{aligned}
N\dot{F\kappa}B &= c5a \cdot I\kappa B\alpha_NF\kappa B - ka1a \cdot NF\kappa B \cdot I\kappa B\alpha + kt2a \cdot pI\kappa B\alpha_NF\kappa B - \\
&- ki1 \cdot NF\kappa B + c5e \cdot I\kappa B\epsilon_NF\kappa B - ka1e \cdot I\kappa B\epsilon \cdot NF\kappa B + \\
&+ kt2e \cdot pI\kappa B\epsilon_NF\kappa B + kd1a \cdot I\kappa B\alpha_NF\kappa B + kd1e \cdot I\kappa B\epsilon_NF\kappa B + \\
&+ ke1 \cdot NF\kappa B \qquad \text{(S1)} \\
nN\dot{F\kappa}B &= -ka1a \cdot kv \cdot nI\kappa B\alpha \cdot nNF\kappa B - ka1e \cdot kv \cdot nI\kappa B\epsilon \cdot nNF\kappa B + \\
&+ ka1e \cdot kv \cdot nI\kappa B\epsilon \cdot nNF\kappa B + kd1a \cdot nI\kappa B\alpha_NF\kappa B - ke1 \cdot nNF\kappa B + \\
&+ c5a \cdot nI\kappa B\alpha_NF\kappa B + c5e \cdot nI\kappa B\epsilon_NF\kappa B + kd1e \cdot nI\kappa B_NF\kappa B + \\
&+ ki1 \cdot NF\kappa B \qquad \text{(S2)} \\
\dot{A20} &= c2 \cdot tA20 - c4 \cdot A20 \qquad \text{(S3)} \\
t\dot{A20} &= c1 \cdot G_{A20}(t) - c3 \cdot tA20 \qquad \text{(S4)} \\
I\dot{\kappa B}\alpha &= -kc1a \cdot IKKa \cdot I\kappa B\alpha - ka1a \cdot I\kappa B\alpha \cdot NF\kappa B + c2a \cdot tI\kappa B\alpha - \\
&- c4a \cdot I\kappa B\alpha - ki3a \cdot I\kappa B\alpha + ke3a \cdot nI\kappa B\alpha + kd1a \cdot I\kappa B\alpha_NF\kappa B \qquad \text{(S5)} \\
tI\dot{\kappa B}\alpha &= c1a \cdot G_{I\kappa B\alpha}(t) - c3a \cdot tI\kappa B\alpha \qquad \text{(S6)} \\
nI\dot{\kappa B}\alpha &= -ka1a \cdot kv \cdot nI\kappa B\alpha \cdot nNF\kappa B + ki3a \cdot I\kappa B\alpha - ke3a \cdot nI\kappa B\alpha + \\
&+ kd1a \cdot nI\kappa B\alpha_NF\kappa B \qquad \text{(S7)} \\
I\kappa B\dot{\alpha_N}F\kappa B &= ka1a \cdot I\kappa B\alpha \cdot NF\kappa B - c5a \cdot I\kappa B\alpha_NF\kappa B - kd1a \cdot I\kappa B\alpha_NF\kappa B + \\
&+ ke2a \cdot nI\kappa B\alpha_NF\kappa B - kc2a \cdot IKKa \cdot I\kappa B\alpha_NF\kappa B \qquad \text{(S8)} \\
nI\kappa B\dot{\alpha_N}F\kappa B &= ka1a \cdot kv \cdot nI\kappa B\alpha \cdot NF\kappa B - ke2a \cdot nI\kappa B\alpha_NF\kappa B - \\
&- kd1a \cdot nI\kappa B\alpha_NF\kappa B - c5a \cdot nI\kappa B\alpha_NF\kappa B \qquad \text{(S9)} \\
pI\dot{\kappa B}\alpha &= kc1a \cdot IKKa \cdot I\kappa B\alpha - kt1a \cdot pI\kappa B\alpha \qquad \text{(S10)} \\
pI\kappa B\dot{\alpha_N}F\kappa B &= kc2a \cdot IKKa \cdot I\kappa B\alpha_NF\kappa B - kt2a \cdot pI\kappa B\alpha_NF\kappa B \qquad \text{(S11)} \\
I\dot{\kappa B}\epsilon &= -kc1e \cdot IKKa \cdot I\kappa B\epsilon - ka1e \cdot I\kappa B\epsilon \cdot NF\kappa B + c2e \cdot tI\kappa B\epsilon - \\
&- c4e \cdot I\kappa B\epsilon - ki3e \cdot I\kappa B\epsilon + ke3e \cdot nI\kappa B\epsilon + kd1e \cdot I\kappa B\epsilon_NF\kappa B \qquad \text{(S12)} \\
tI\dot{\kappa B}\epsilon &= c1e \cdot G_{I\kappa B\epsilon}(t - T_D) - c3e \cdot tI\kappa B\epsilon \qquad \text{(S13)} \\
nI\dot{\kappa B}\epsilon &= -ka1e \cdot kv \cdot nI\kappa B\epsilon \cdot nNF\kappa B + ki3e \cdot I\kappa B\epsilon - ke3e \cdot nI\kappa B\epsilon + \\
&+ kd1e \cdot nI\kappa B\epsilon_NF\kappa B \qquad \text{(S14)}
\end{aligned}$$

$$\dot{I\kappa B\epsilon\_NF\kappa B} = ka1e \cdot I\kappa B\epsilon \cdot NF\kappa B - c5e \cdot I\kappa B\epsilon\_NF\kappa B - kd1e \cdot I\kappa B\epsilon\_NF\kappa B + +ke2e \cdot nI\kappa B\epsilon\_NF\kappa B - kc2e \cdot IKKa \cdot I\kappa B\epsilon\_NF\kappa B \quad (S15)$$

$$\dot{nI\kappa B\epsilon\_NF\kappa B} = ka1e \cdot kv \cdot nI\kappa B\epsilon \cdot NF\kappa B - ke2e \cdot nI\kappa B\epsilon\_NF\kappa B - -kd1e \cdot nI\kappa B\epsilon\_NF\kappa B - c5e \cdot nI\kappa B\epsilon\_NF\kappa B \quad (S16)$$

$$\dot{pI\kappa B\epsilon} = kc1e \cdot IKKa \cdot I\kappa B\epsilon - kt1e \cdot pI\kappa B\epsilon \quad (S17)$$

$$\dot{pI\kappa B\epsilon\_NF\kappa B} = kc2e \cdot IKKa \cdot I\kappa B\epsilon\_NF\kappa B - kt2e \cdot pI\kappa B\epsilon\_NF\kappa B \quad (S18)$$

$$\dot{IKKKa} = TR \cdot kr \cdot (KN - IKKK) - kri \cdot IKKKa - kaA20 \cdot A20 \cdot IKKKa \quad (S19)$$

$$\dot{IKKn} = kp \cdot \frac{kbA20}{kbA20 + A20} \cdot (KNN - IKKn - IKKa - IKKi) - -ka \cdot IKKKa \cdot IKKn \quad (S20)$$

$$\dot{IKKa} = ka \cdot IKKK - ki \cdot IKKa \quad (S21)$$

$$\dot{IKKi} = ki \cdot IKKa - kii \cdot IKKi \quad (S22)$$

Stochastic processes

The state of each gene promoter could be either on or off depending on whether NF-κB molecule is bound or unbound to it. Since each of the genes in the model has two independent homologs, the state of transcriptional activity of each gene can be described by $G(t) \in \{0, 1, 2\}$. Furthermore, because of transcriptional delay of IκBϵ proteins, we must consider the delayed state of transcriptional activity, $G_{I\kappa B\epsilon}(t-T_D) \in \{0, 1, 2\}$.

To calculate the state of transcriptional activity of each gene, we must consider the binding and dissociation propensities of NF-κB. The binding propensity, r^b, is assumed to be proportional to the nuclear NF-κB concentration, while the dissociation propensity, r^d, is proportional to the nuclear IκB protein concentration:

$$r^b(t) = q_1 \cdot nNF\kappa B(t, G(t)) \quad (S23)$$

$$r^d(t) = q_{2a} \cdot nI\kappa B\alpha(t, G(t)) + q_{2e} \cdot nI\kappa B\epsilon(t, G(t)) \quad (S24)$$

The total propensity for the occurrence of any binding or dissociation event can be

described by

$$r(t) = r^b_{A20} \cdot (2 - G_{A20}(t)) + r^b_{I\kappa B\alpha} \cdot (2 - G_{I\kappa B\alpha}(t)) + r^b_{I\kappa B\epsilon} \cdot (2 - G_{I\kappa B\epsilon}(t)) + r^d_{A20} \cdot G_{A20}(t) + r^d_{I\kappa B\alpha} \cdot G_{I\kappa B\alpha}(t) + r^d_{I\kappa B\epsilon} \cdot G_{I\kappa B\epsilon}(t) \quad \text{(S25)}$$

Using Fortran 90, we eployed the following algorithm (Paszek *et al*, 2010) to compute the time evolution of the system. For all simulations, we ran this algorithm until steady state values were reached and saved scheduled delayed reactions and their times before perturbing the system.

1. At $t = t_0$, initialize state of discrete variables, $G(t_0)$. Set $G_{I\kappa B\epsilon}(t) = 0$ for $t \in [t_0 - T_D, t_0)$.

2. Select two random numbers, p_1 and p_2 from the uniform distribution on $(0, 1)$.

3. Using fifth and sixth order Runge-Kutta solver, dverk, evaluate the system of model ODEs until time $t + \tau$, where

   $$ln(p_1) + \int_t^{t+\tau} r(s)ds = 0.$$

   Incorporate any scheduled delayed reactions in the time interval $[t, t + \tau)$.

4. Choose which stochastic reaction j is to occur at $t + \tau$ by finding j such that

   $$\sum_{i=1}^{j-1} r_i(t + \tau) < p_2 \cdot r(t + \tau) \leq \sum_{i=1}^{j} r_i(t + \tau)$$

   where $r_i, i = 1, ..., 6$ are individual reaction propensities.

5. Update discrete variable states G_{A20}, $G_{I\kappa B\alpha}$, and $G_{I\kappa B\epsilon}$.

6. Replace t with $t + \tau$ and repeat from 2
